# P2X7R of synovial fibroblasts is a potential therapeutic target associated with refractory rheumatoid arthritis

**DOI:** 10.1126/sciadv.adw9543

**Published:** 2026-05-22

**Authors:** Peishi Rao, Shanzhao Jin, Shibai Xiao, Yinchao Ma, Jing Li, Yuhua Liao, Ziye Wang, Yundi Tang, Xuanlin Cai, Xingyue Zeng, Yixiang Hong, Xiaocheng Wang, Jiaxin He, Shenjie Ma, Junyi Jiang, Wenjuan Zhang, Baozhen Zhang, Ru Li, Liang Zhang, Chuanhui Xu, Qingping Jin, Qingwen Wang, Ming Chu, Xiaolin Sun, Zhanguo Li

**Affiliations:** ^1^ Department of Rheumatology and Immunology, Peking University People’s Hospital & Beijing Key Laboratory Non-invasive Diagnosis and Immunotherapy of Rheumatic Diseases, Beijing, China.; ^2^ Department of Rheumatology, Zhongshan Hospital (Xiamen), Fudan University, Xiamen, China.; ^3^ Department of Rheumatism and Immunology, Peking University Shenzhen Hospital, Shenzhen, China.; ^4^ NHC Key Laboratory of Medical Immunology (Peking University), School of Basic Medical Sciences, Peking University, Beijing, China.; ^5^ Zhejiang Jinhua Conba Bio-Pharm. Co. Ltd., Jinhua, China.; ^6^ State Key Laboratory of Medical Proteomics, Beijing Proteome Research Center, National Center for Protein Sciences (Beijing), Beijing Institute of Lifeomics, Beijing, China.; ^7^ General Practice Department, Beijing Jishuitan Hospital, Beijing, China.; ^8^ Department of Nephrorheumatology, Beijing Jishuitan Hospital Guizhou Hospital, Beijing, China.; ^9^ Department of Orthopedics, Beijing Jishuitan Hospital, Fourth Clinical College of Peking University, Beijing, China.; ^10^ Department of Rheumatology, Allergy and Immunology, Tan Tock Seng Hospital, Jln Tan Tock Seng, Singapore 308433, Singapore.; ^11^ Lee Kong Chian School of Medicine, Nanyang Technological University, Singapore 308232, Singapore.; ^12^ Peking-Tsinghua Center for Life Sciences, Peking University, Beijing, China.; ^13^ State Key Laboratory of Natural and Biomimetic Drugs, School of Pharmaceutical Sciences, Peking University, Beijing, China.

## Abstract

Rheumatoid arthritis (RA) is a chronic autoimmune disease characterized by persistent synovitis. Synovial fibroblasts (SF) are the predominant cellular components in the inflamed synovium. Recent studies have shown increased expression of P2X7 receptor (P2X7R) in RA SF. However, its precise expression patterns and its contribution to inflammatory arthritis remain unclear. We demonstrate that P2X7R is robustly expressed in RA synovium, correlating with increased synovitis and systemic inflammation. Single-cell transcriptome analysis indicated that P2X7R was enriched in specific SF associated with refractory RA. Then, we applied a highly selective human P2X7R antagonist, EVT-401, which promoted apoptosis and induced cell cycle arrest in RA SF, reduced the production of proinflammatory and joint-destructive mediators, and mitigated aggressive phenotypes. Furthermore, we demonstrated that EVT-401 markedly reduced synovial inflammation and arthritis severity in a nonhuman primate model of autoimmune arthritis. Together, these findings suggest that P2X7R is involved in synovial inflammation and its inhibition may represent a potential therapeutic strategy for refractory RA.

## Teaser

Targeting P2X7R in synovial fibroblasts offers a promising strategy for treating refractory rheumatoid arthritis.

## INTRODUCTION

Rheumatoid arthritis (RA) is a systemic autoimmune disorder characterized by inflammation in multiple joints, affecting ~1% of the global population (*1*). This chronic condition frequently results in joint dysfunction and increases the risk of disability (*2*). Despite advancements in targeted therapies, including tumor necrosis factor (TNF) inhibitors, interleukin-6 (IL-6) receptor blockers, B cell–targeting agents (anti-CD20), and Janus kinase inhibitors (*3*), nearly 40% of patients fail to respond to a given drug, and 5 to 20% develop resistance to all available treatments (*4*). This often results in an irreversible and disabling disease trajectory, underscoring the urgent need to identify previously unidentified therapeutic targets for multidrug-resistant refractory RA.

Erosive synovitis, which causes joint cartilage and bone destruction, is a hallmark of RA (*5*). Synovial fibroblasts (SF) are the predominant cellular component in the inflamed synovium of patients with RA (*6*). Recent single-cell omics studies have revealed significant functional heterogeneity among SF subsets in patients with RA (*7*–*11*). Lining SF primarily drive invasive pannus formation and joint destruction, while sublining SF produce proinflammatory cytokines and chemokines (*11*). In-depth histological and transcriptomic studies have further demonstrated the clinical relevance of RA synovial cellular compositions in predicting therapeutic responses to targeted treatments (*12*–*16*). For instance, the R4RA study revealed that synovium from patients with refractory RA and multidrug resistance was characterized by minimal immune cell infiltration and a predominance of SF (*17*). Transcriptomic analyses further linked an enriched SF gene expression signature with drug resistance in RA (*18*, *19*). Zhang *et al.* (*20*) proposed the concept of cell-type abundance phenotypes (CTAPs) defined by the enrichment of specific cell types. Among these, CTAP–fibroblast-rich subtype (F), characterized by the highest prevalence of SF, was identified as the multidrug-resistant type. Collectively, these findings suggest that an enrichment of SF coinciding with limited immune cell infiltration was associated with poor treatment responses in refractory RA. Developing therapeutic agents targeting SF could therefore improve outcomes in patients with multidrug-resistant RA.

The P2X7 receptor (P2X7R) is a trimeric ligand-gated cation channel that binds to adenosine 5′-triphosphate (ATP), leading to channel opening and subsequent Na^+^ and Ca^2+^ influx, as well as depolarization of the resting membrane potential (*21*). Up-regulation of P2X7R expression and activation has been implicated in the progression of various autoimmune disorders (*22*–*24*), driving the release of inflammatory mediators and influencing cell proliferation and apoptosis (*25*, *26*). Given its role in immune modulation, P2X7R has emerged as a potential therapeutic target in RA (*27*). Studies have suggested that P2X7R promotes the inflammatory response in RA by influencing T helper 17 cell (Th17 cell) differentiation (*28*), promoting IL-1β production in macrophages (*29*), and modulating neutrophil apoptosis (*30*). However, several clinical trials (NCT00520572 and NCT00628095) targeting P2X7R for RA treatment have failed in recent years, suggesting that the precise contribution of P2X7R to RA pathogenesis and its therapeutic potential remain inadequately understood, necessitating further investigation.

In this study, we found that P2X7R was significantly up-regulated in RA synovium and correlated with both local synovitis and systemic inflammation. Single-cell transcriptome analysis showed that P2X7R expression was enriched in specific SF subpopulations associated with refractory RA. Building on these findings, we showed that EVT-401 is a highly selective human P2X7R (hP2X7R) inhibitor, which reversed inflammatory phenotypes in vitro by modulating P2X7R-associated pathogenic pathways, promoting apoptosis, and inducing cell cycle arrest in RA SF. In addition, it reduced the production of proinflammatory and joint-destructive mediators. In a preclinical nonhuman primate (NHP) autoimmune arthritis model, EVT-401 significantly reduced synovial inflammation and arthritis severity, demonstrating both efficacy and safety. These results revealed that P2X7R might contribute to multidrug-resistant synovial inflammation and its inhibition could be a potential therapy for refractory RA.

## RESULTS

### Overexpression of synovial P2X7R associated with inflammation in RA

**Fig. 1.**
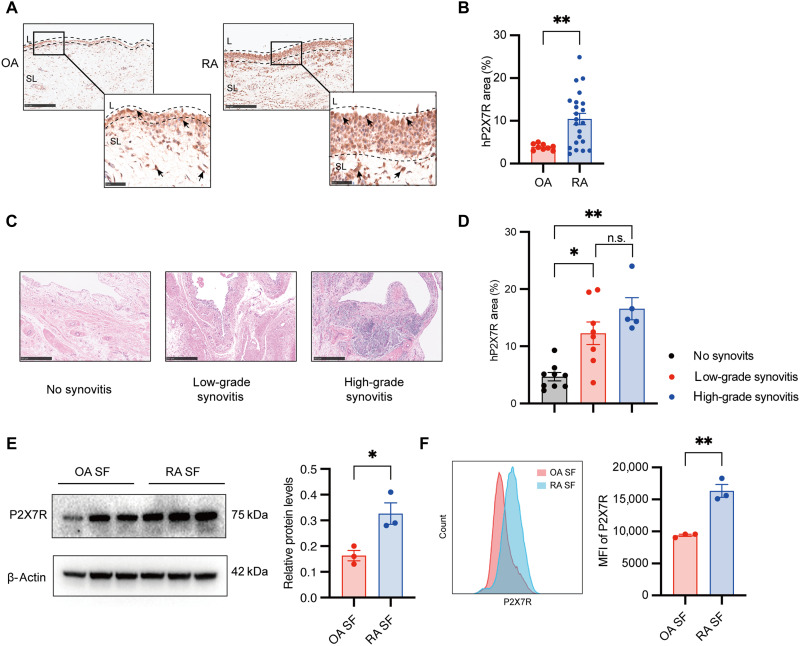
P2X7R was highly expressed in synovial tissues and SF in patients with RA and was correlated with systemic inflammation. (**A**) IHC staining of P2X7R in OA and RA synovial tissues. Representative images and quantitative analysis are shown. Scale bars, 250 μm (overview) and 50 μm (zoomed-in images). The black border indicates the lining layer (L), and the remaining areas represent the sublining layer (SL). Black arrows indicate P2X7R-positive cells. *n* = 10 individuals in the OA group and *n* = 22 individuals in the RA group. (**B**) Comparison of P2X7R expression in synovial tissue between patients with RA and OA. (**C** and **D**) Representative H&E images of synovial sections from patients with RA and the expression of P2X7R in different grades of synovitis. Scale bars, 500 μm. (**E**) Representative Western blots and quantitative analysis of P2X7R protein expression. (**F**) Fluorescence-activated cell sorting (FACS) analysis and statistical analysis of P2X7R expression in OA and RA SF. Data are shown as the mean ± SEM and are derived from primary SF (passages 4 to 7) derived samples of three independent patients with RA and at least two independent experiments. Statistical significance was determined using Mann-Whitney test [(B), (E), and (F)] and Kruskal-Wallis test with Dunn’s post hoc test (D). *P < 0.05, **P < 0.01, and n.s., not significant.

The expression profile of P2X7R in RA synovial tissue and its contribution to synovitis remain incompletely characterized. To investigate this, we collected synovial tissue samples from patients with RA undergoing joint replacement surgery and evaluated P2X7R expression by immunohistochemistry (IHC), alongside histopathological assessment using hematoxylin and eosin (H&E) staining. The demographic and clinical characteristics of the patient cohort were summarized in table S1. IHC analysis revealed pronounced synovial lining hyperplasia in RA samples, with P2X7R expression predominantly localized to stromal cells within the expanded lining layer and discrete regions of the sublining area (Fig. 1A). Quantitative analysis further demonstrated that P2X7R expression was significantly elevated in RA synovial tissues compared to osteoarthritis (OA) controls (*P* = 0.0046; Fig. 1B).

A positive correlation was observed between synovial P2X7R expression and serum C-reactive protein (CRP) levels [*P* = 0.0441, correlation coefficient (*r*) = 0.4434; fig. S1]. However, this association was largely driven by a single outlier samples with exceptionally high CRP levels. Correlations between P2X7R expression and other clinical parameters—including erythrocyte sedimentation rate, rheumatoid factor, Disease Activity Score in 28 Joints (DAS28), and anti–cyclic citrullinated peptide antibody—were also assessed but showed no significant associations (fig. S1).

Subsequently, synovitis severity was quantified using the Krenn synovitis scoring system (*31*). On the basis of the synovitis scores, synovial samples were categorized into “no synovitis,” “low-grade synovitis,” and “high-grade synovitis” groups (Fig. 1C and table S2). P2X7R expression was significantly higher in synovial samples with both low-grade and high-grade synovitis compared to samples without synovitis (*P* = 0.019 and *P* = 0.001, respectively; Fig. 1D). While expression levels tended to be higher in high-grade than in low-grade synovitis, this difference did not reach statistical significance (*P* = 0.19; Fig. 1D).

Next, SF were isolated and cultured from inflamed synovium of patients with RA or OA. Consistent with the tissue-level findings, P2X7R expression in RA SF was significantly higher than in OA fibroblasts, as confirmed by both Western blotting and flow cytometry (Fig. 1, E and F). These findings demonstrate that P2X7R is markedly up-regulated in the inflamed synovial tissues and fibroblasts of patients with RA. Its expression correlates positively with synovitis severity, highlighting its potential role in RA pathogenesis.

### Preferential expression of P2X7R in RA SF

**Fig. 2.**
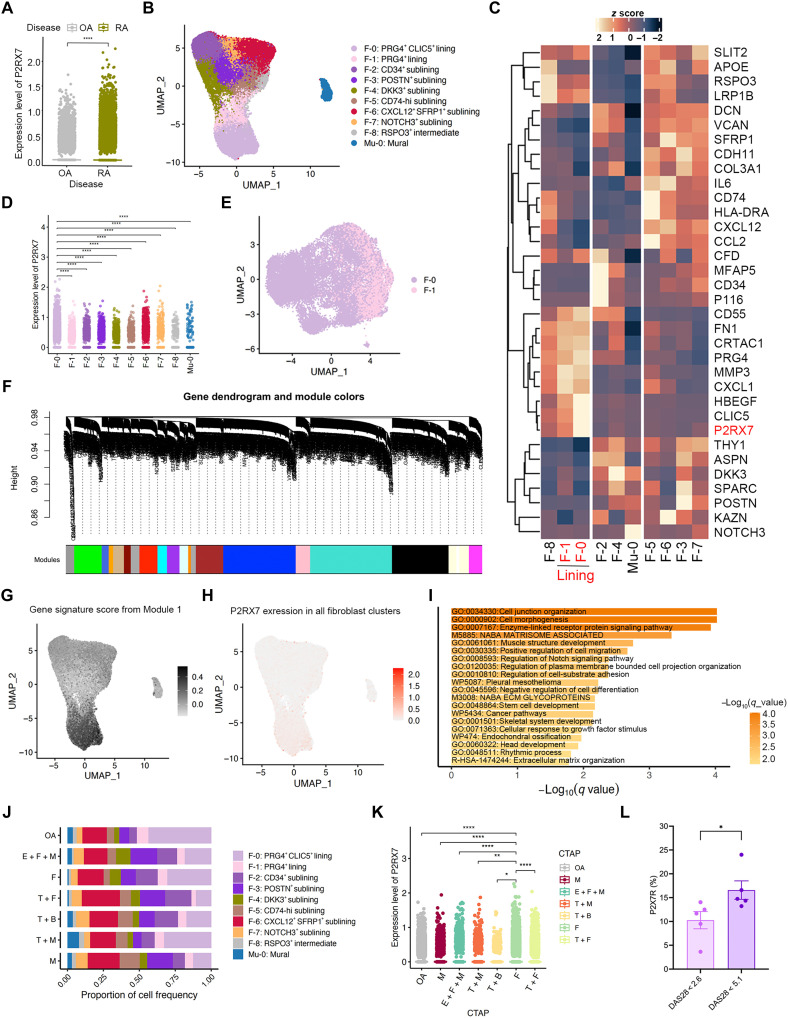
*P2X7R* was enriched in lining fibroblasts and was associated with poor therapeutic response. (**A**) Scatter plot comparing the expression of *P2RX7* between the RA and OA groups. (**B**) Uniform manifold approximation and projection (UMAP) plot showing the distribution of fibroblast cells across identified clusters. (**C**) Heatmap displaying normalized gene expression values (*z* scores) of fibroblast signature genes across subclusters. Hierarchical clustering was applied to rows (genes) to highlight expression patterns. (**D**) Scatter plot comparing the expression of *P2RX7* between F-0 and other subclusters. (**E**) UMAP visualization of single-cell transcriptomes, with clusters annotated as F-0 and F-1. (**F**) Gene dendrogram displaying hierarchical clustering of genes. Different colors indicate distinct modules, with black representing module 1. (**G**) UMAP plot showing the enrichment of Module 1 expression in fibroblast clusters. (**H**) UMAP plot depicting the expression levels of *P2RX7* in fibroblast clusters, with red indicating high expression and gray representing low expression. (**I**)The top 20 pathways enriched by Module 1 genes (Metascape) were plotted and ranked by −log_10_ (*q*) with Benjamini-Hochberg false discovery rate (FDR). (**J**) The proportions of fibroblast subclusters across CTAP groups were shown. CTAPs had been categorized by dominant cell types: (i) endothelial + fibroblast + myeloid (E + F + M), (ii) fibroblasts (F), (iii) T cells + fibroblasts (T + F), (iv) T + B cells (T + B), (v) T + myeloid cells (T + M), and (vi) myeloid (M). (**K**) Scatter plot comparing the expression of *P2RX7* between CTAP-F and other CTAP groups. (**L**) Bar plot showing the percentage of P2X7R^+^ cells in synovial tissue (IHC quantification) in patients with RA stratified by DAS28 categories: remission (DAS28 < 2.6) versus high disease activity (DAS28 ≥ 5.1). Dots represent individual patients; bars indicate mean ± SEM. Statistical significance was determined using the two-sided Wilcoxon test [(A), (D), and (K)] and Mann-Whitney test (L). *P < 0.05, ** *P* < 0.01, and ****P < 0.0001.

Given the prominent expression of P2X7R in stromal cells within the synovium, we aimed to further characterize its distribution across SF subsets and evaluate its clinical significance. To achieve this, we analyzed single-cell transcriptomic data from RA synovium, as reported by Zhang *et al.* (syn52297840) (*20*). P2X7R mRNA (*P2RX7*) levels were significantly elevated in SF from patients with RA compared to those from patients with OA (Fig. 2A), in agreement with protein-level observations. Using this dataset, we recapitulated the SF clustering described in Zhang *et al.* work. Synovial stromal cells were broadly categorized into three groups: lining (PRG4^hi^), sublining (THY1^+^PRG4^low^), and mural cells (NOTCH3^+^MCAM^+^, also known as CD146^+^) (Fig. 2B). Evaluation of *P2RX7* expression levels across these subpopulations revealed that the PRG4*^+^*CLIC5^+^ subset (F-0) and the PRG4^+^ subset (F-1), which constitute the synovial lining layer, exhibited significantly higher *P2RX7* expression compared to sublining fibroblast subsets (Fig. 2C). Notably, the F-0 population displayed the highest *P2RX7* expression among all RA SF subsets (Fig. 2D).

To further delineate the gene programs associated with *P2RX7*, we analyzed the lining fibroblast subclusters for coexpression regulatory network analysis (Fig. 2E). Weighted Gene Coexpression Network Analysis (WGCNA) revealed that, in the F-0 and F-1 subsets, *P2RX7* coexpressed with genes in the black module (Module 1) of the gene regulatory network (Fig. 2F).

In RA synovium, *P2RX7* expression was observed to be mainly enriched in myeloid cells and SF (fig. S2, A and B). Although *P2RX7* expression was higher in myeloid cells (fig. S2B), Module 1 gene signature score showed preferential enrichment in SF (fig. S2, C and D), suggesting that this *P2RX7*-associated coexpression module reflects an SF-specific coordinated functional program. Moreover, feature plots revealed that the Module 1 activity was markedly elevated in the F-0/F-1 fibroblast subsets compared with other clusters (Fig. 2G), in line with the distribution pattern of *P2RX7* expression across fibroblast populations (Fig. 2H). This module was enriched for processes related to cell morphogenesis (e.g., *ANK3*, *NTNG2*, and *CDH15*), extracellular matrix (ECM) organization (e.g., *ADAMTSL1*, *COL22A1*, and *OLFML2B*), cellular responses to growth factor stimuli, regulation of chemotaxis (e.g., *HBEGF*, *CSF1*, *EREG*, and *ITGA6*), and the regulation of Notch signaling [e.g., *APOB* and beta-1,3-N-acetylglucosaminyltransferase lunatic fringe (*LFNG*)] (Fig. 2I). Consistently, coexpression network analysis revealed that Module 1 activity correlates with *P2RX7* and is significantly enriched for Notch [*NOTCH1 to 4*, Notch intracellular domain (NICD) processing, and RUNX3 regulation] and cell-cycle programs [transcription factor *E2F2* (*EF2*) and G_2_-M checkpoint], with core hubs including a disintegrin and metalloproteinase domain-containing protein 10 (*ADAM10*), tissue inhibitor of matrix metalloproteinases 3 (*TIMP3*), *LFNG*, and mitogen-activated protein kinase 7 [(*MAPK7*); fig. S3)].

Because Notch signaling drives proliferation and differentiation of pathogenic RA SF (*32*) and growth factor responses have been implicated in RA relapse (*33*), these findings correspond to the pathogenic roles of lining SF in promoting invasive pannus formation and joint destruction. Among the sublining SF, which expressed much lower levels of *P2RX7* than the lining SF, *P2RX7* was still relatively higher expressed in the DKK3^+^ subset (F-4) and NOTCH3^+^ subset (F-7) (Fig. 2C). These subsets were characterized by gene enrichment in Notch signaling (e.g.,*NOTCH3*), proinflammatory cytokines and chemokines (e.g., *IL6*, *CXCL12*, and *CCL2*), antigen presentation (e.g. *HLA-DRA*), and markers associated with refractory RA (e.g., *DKK3* and *CD34*) (*19*), suggesting their contributions to RA inflammation and autoimmune responses. In summary, our findings highlight that P2X7R mRNA (*P2RX7*) is predominantly expressed in key pathogenic RA SF subsets, underscoring its potential roles in driving the invasive, destructive, and proinflammatory effects of the RA synovium.

### Up-regulation of P2X7R in SF was predominant in refractory RA

Previous studies have shown that the cellular composition and molecular signatures of RA synovium are associated with the treatment responses in patients with RA receiving various targeted biologic therapies (*34*). The R4RA study demonstrated that patients with refractory RA predominantly presented with a fibroid, pauci-immune synovial pathotype. This pathotype was characterized by limited immune cell infiltration and a predominance of SF (*17*). Consistent with the R4RA study, Zhang *et al.* (*20*) used single-cell RNA sequencing (RNA-seq) analysis of synovial tissue to define six distinct CTAPs. Among them, CTAP-F, characterized by the highest prevalence of SF, was identified as the multidrug-resistant phenotype. This CTAP-F gene signature was further validated in the R4RA cohort, demonstrating its potential to identify patients with multidrug-resistant RA. Given the association between elevated *P2RX7* expression and the refractory RA synovial markers *DKK3* and *CD34*, we evaluated *P2RX7* expression in SF across different CTAPs to explore its link with treatment responses. Further analysis identified nine distinct SF subpopulations, with their proportions varying across the different CTAP groups (Fig. 2J). We found that the *P2RX7*-overexpressing lining fibroblast subsets, F-0 and F-1, were expanded in CTAP-F (Fig. 2J).

Furthermore, *P2RX7* levels were highest in CTAP-F among the six CTAPs (Fig. 2K), suggesting that elevated *P2RX7* was associated with the treatment resistance risk indicated by CTAP-F. To validate this finding, we analyzed synovial P2X7R levels in our cohort after stratifying patients by posttreatment disease activity, which was assessed using DAS28. According to the established European League Against Rheumatism (EULAR) response criteria (*35*), DAS28 < 2.6 is widely accepted to indicate clinical remission, whereas a score > 5.1 reflects moderate-to-high disease activity. Consistent with these categories, our results revealed a significant elevation of synovial P2X7R levels in patients with inadequate responses to conventional treatment, who exhibited higher DAS28 (Fig. 2L). These findings further reinforce the association between elevated *P2RX7* expression and resistance to multidrug therapies in RA.

### EVT-401 is a potent and highly selective hP2X7R antagonist

**Fig. 3.**
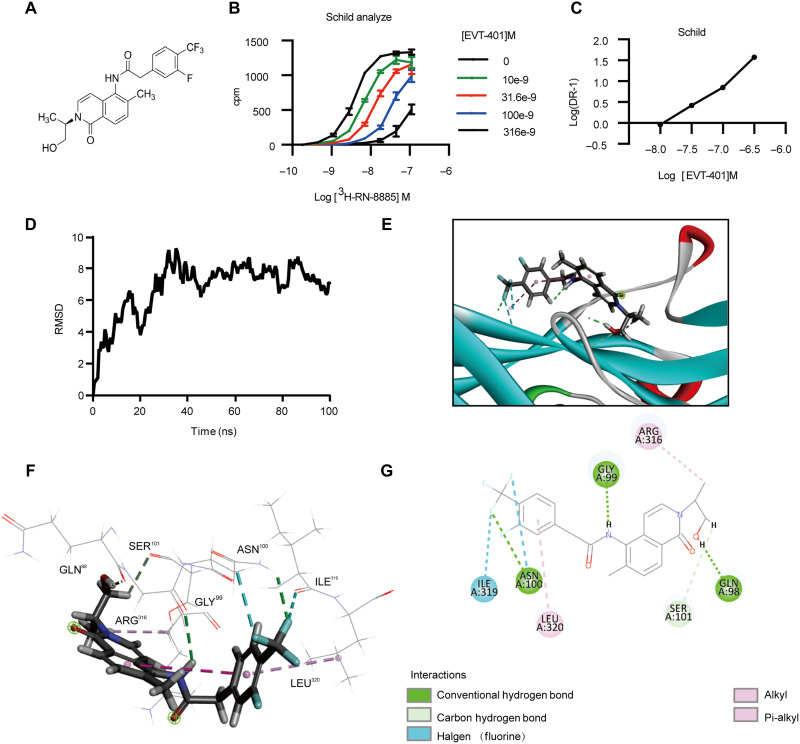
The binding property of EVT-401 with hP2X7R. (**A**) The structure of compound EVT-401. (**B** and **C**) Schild analysis (KB) for determining EVT-401’s binding affinity for hP2X7R using ^3^H-RN-0278885 radioligand binding assay. (**D**) Molecular dynamics simulation of hP2X7R in complex with EVT-401. Stability was analyzed by root mean square deviation (RMSD) versus time (in nanoseconds). (**E**) Binding features of EVT-401 with hP2X7R. Secondary structural elements are depicted as ribbons (coils for α helices; arrows for β sheets). Colors represent secondary structures: α helices in red, β sheets in sky blue, and loops in green. (**F**) Molecular interactions of EVT-401 with the residues of hP2X7R. The residues are shown as sticks and labeled in black. (**G**) Electrostatic interactions shown as dashed lines, with π-π, π-alkyl, halogen, and hydrogen bonds colored purple, pink, blue, and green, respectively.

Given the significant elevation of P2X7R in patients with treatment-resistant RA, we conducted further investigations targeting P2X7R to evaluate its potential therapeutic efficacy. EVT-401, a human-selective small molecular antagonist of P2X7R [2- (3-fluoro-4- (trifluoromethyl)phenyl)-N- (2- (1-hydroxypropan-2-yl)-6-methyl-1-oxo-1,2-dihydroisoquinolin-5-yl)acetamide] was applied in our study (Fig. 3A). The characteristics and chemical synthesis route of EVT-401 were described in fig. S4. The in vitro stability data, mean plasma concentration and pharmacokinetic parameters of EVT-401 were shown in tables S3 to S6. To assess the binding affinity of EVT-401 to hP2X7R, we radiolabeled EVT-401 with tritium and performed competitive binding assays to determine the equilibrium dissociation constant (*K*_i_) and conduct Schild analysis. The binding affinity was determined to be 7.6 nM [95% confidence interval (CI): 6.3 to 9.1 nM] by competitive binding and 12.1 nM (95% CI: 11.8 to 12.3 nM) by Schild analysis (Fig. 3, B and C).

P2X7R function was evaluated by measuring calcium influx, given its role as a nonselective cation channel. For these functional assays, we used the astrocytoma cell line 1321N1, which lacks an endogenous calcium influx response to P2X7R agonists due to the absence of endogenous P2 receptor expression. When transfected with human or murine P2X7R, or other P2 receptors such as P2X1 or P2X2, this cell line will show a calcium influx signaling after stimulated with 2′(3′)-O-(4-benzoylbenzoyl)adenosine 5′-triphosphate (BzATP). As shown in table S7 and fig. S5 (A and B), EVT-401 differentially inhibited P2X7R-mediated calcium influx in 1321N1 cells expressing human and rat P2X7R. The median inhibitory concentration (IC_50_) for the hP2X7R was 10.0 ± 2.0 nM (*n* = 3), while for the rat P2X7R, it was 220 ± 19.4 nM (*n* = 4), indicating that EVT-401 was 22.0 times more potent against the hP2X7R than the rat P2X7R, confirming its higher ability to functionally inhibit hP2X7R, which is high human selectivity. Furthermore, the calcium influx signals from four 1321N1 cell lines individually expressing four different P2X7-related P2 receptors (P2X1R, P2X2R, P2X3R, and P2X4R) were tested. This result showed that no calcium signal reduction was observed in all these four cell lines when treated with different concentrations of EVT-401, which indicated that EVT-401 could not inhibit the functions of these P2X7R-related receptors, reinforcing its functional specificity on its designated target, P2X7R. Notably, if EVT-401 could also affect other targets to alter the calcium influx in this human cell line, then it would induce an obvious calcium signal change in these cell lines not expressing P2X7R. As we did not observe such phenomena, EVT-401 might not inhibit calcium influx by affecting other target moleculars except P2X7R, at least in these human cell lines (table S7 and fig. S5C).

To further validate these findings and assess the binding characteristics of EVT-401, we conducted molecular docking analyses. As shown in Fig. 3 (D to G), EVT-401 displayed a low binding energy of −37.4239 kcal/mol, suggesting highly stable binding. The simulation indicated that EVT-401 may interact with hP2X7R residues Gly^99^, Gln^98^, Ser^101^, Asn^100^, Arg^316^, Ile^319^, and Leu^320^. Collectively, these data support EVT-401 as a potent and highly selective antagonist for hP2X7R.

### EVT-401 inhibited the proliferation of RA SF by inducing G_1_ cell cycle arrest

**Fig. 4.**
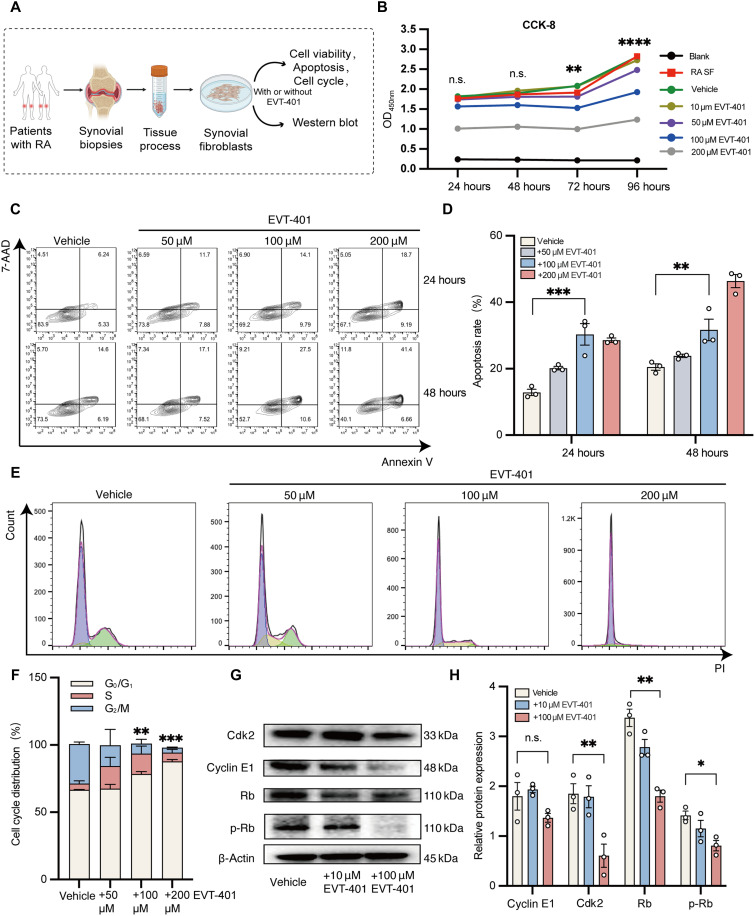
EVT-401 induced G_1_ cell cycle arrest in RA SF. (**A**) Schematic diagram illustrating the design of the in vitro study. (**B**) CCK-8 analysis of cell viability in RA SF at the indicated time points under different concentrations of EVT-401 treatment (*n* = 6 per group, and all significance comparisons were made between the vehicle group and the 100 μM EVT-401 group). (**C** and **D**) Apoptosis analysis of RA SF treated with 50, 100, and 200 μM EVT-401 for 24 or 48 hours. The proportion of apoptotic cells was quantified using flow cytometry with annexin V/7-aminoactinomycin D (7-AAD) staining (*n* = 3 per group). (**E** and **F**) Representative flow cytometry plots showing the cell cycle distribution of RA SF after treatment with varying doses of EVT-401 for 24 hours (*n* = 3 per group). (**G** and **H**) Western blot analysis of cell cycle–related proteins, including Cdk2, Cyclin E1, p-Rb, and Rb, in RA SF following EVT-401 treatment (*n* = 3 per group). Data are shown as the mean ± SEM and are derived from three cell lines and at least two independent experiments. Statistical significance was determined using a two-way analysis of variance (ANOVA) with Tukey’s multiple-comparison test (B), Mann-Whitney test (D and H), or a two-way ANOVA for the G_0_/G_1_ distribution in the EVT-401 group (F) compared with the vehicle group. *P < 0.05, **P < 0.01, and ***P < 0.001 [EVT-401 group: RA SF + EVT-401; vehicle: RA SF + 0.1% dimethyl sulfoxide (DMSO)]. Created in BioRender. Shi, Y. (2026) https://BioRender.com/88xnz97.

Abnormal proliferation of SF in RA leads to synovial hyperplasia and hypertrophy, contributing to pannus formation and cartilage destruction (*5*). To investigate whether P2X7R blockade could inhibit the abnormal proliferation, we isolated and cultured SF from the inflamed synovium of patients with RA (Fig. 4A). We assessed the viability and proliferation of RA SF treated with EVT-401 using the cell counting kit-8 (CCK-8) assay. The results showed that 100 μM EVT-401 partially inhibited the proliferation of RA SF after 72 hours of incubation without significant inhibitive effects on SF proliferation at 24 or 48 hours. Meanwhile, concentrations of EVT-401 below 100 μM did not show significant inhibitory effects on cell proliferation at 24, 48, or 72 hours (Fig. 4B). To explore the underlying mechanism, we observed that 100 μM EVT-401 promoted cell apoptosis after 1 and 2 days of incubation (Fig. 4, C and D). Further analysis of the cell cycle revealed that 100 μM EVT-401 induced G_0_/G_1_ cell cycle arrest compared with vehicle (Fig. 4, E and F). In addition, Western blot analysis of cell cycle–related proteins revealed that levels of G_0_/G_1_ phase–related proteins, such as cyclin-dependent kinase 2 (Cdk2), retinoblastoma protein (Rb), and phosphorylated Rb (p-Rb), were significantly reduced in EVT-401–treated SF (Fig. 4, G and H). In summary, the P2X7R antagonist effectively suppressed the growth of RA SF by inhibiting cell cycle progression and inducing apoptosis.

### EVT-401 interferes with the inflammatory phenotypes of RA SF

**Fig. 5.**
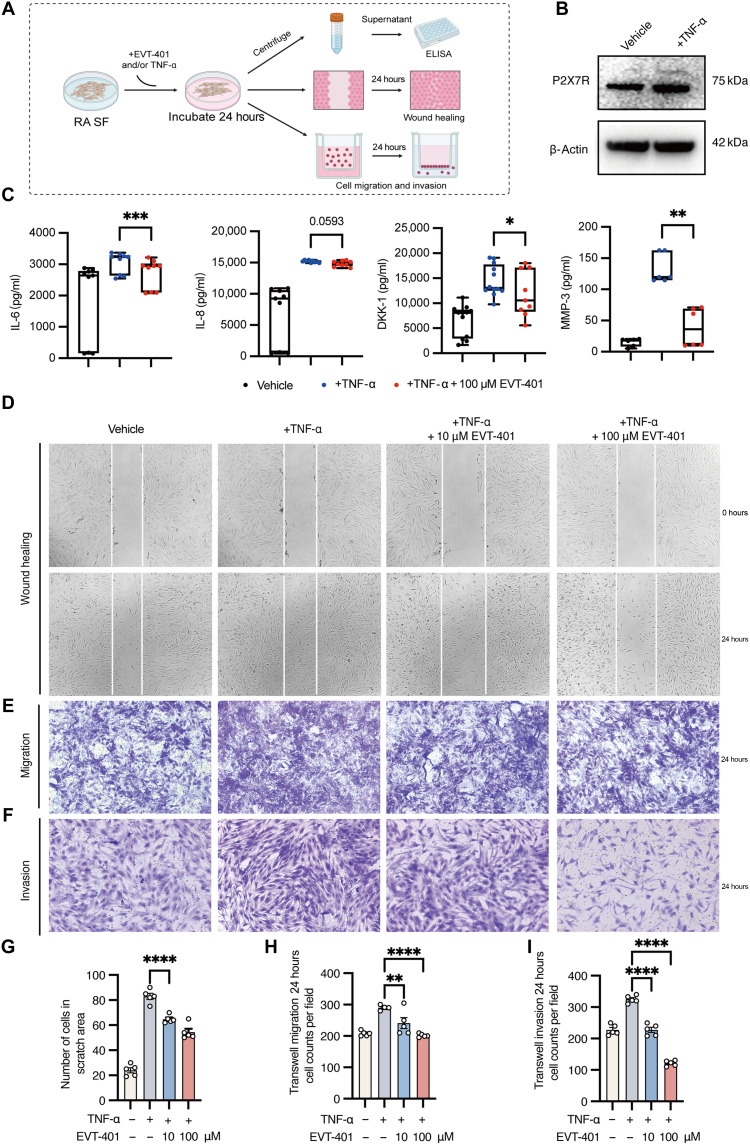
Effects of EVT-401 on aggressive phenotypes of SF. (**A**) Schematic diagram of the experimental design. (**B**) Representative Western blots showing P2X7R protein levels in RA SF after TNF-α stimulation. (**C**) The release of IL-6, IL-8, DKK-1, and MMP-3 in culture supernatants was measured using specific ELISA assays. (**D** and **G**) Effect of EVT-401 on the scratch assay. (**E** and **H**) Effect of EVT-401 on migration of RA SF. (**F** and **J**) Effect of EVT-401 on invasion of RA SF. Representative images are shown (*n* = 5 in each) (original magnification: ×100). Data are shown as the mean ± SEM and are derived from three cell lines and at least two independent experiments. Statistical significance was determined using Mann-Whitney test (C) and one-way ANOVA test (**G** to **I**). *P < 0.05, **P < 0.01, and ***P < 0.001 (EVT-401 group: RA SF + TNF-α + EVT-401, TNF-α group: RA SF + TNF-α + 0.1% DMSO, and vehicle: RA SF + 0.1% DMSO). Created in BioRender. Shi, Y. (2026) https://biorender.com/oxyfttv.

Exogenous TNF-α is commonly used to simulate the inflammatory microenvironment characteristic of RA. SF-mediated inflammation plays a critical role in the pathogenesis of RA. To investigate the anti-inflammatory effects of the P2X7R antagonist, we exposed RA SF to TNF-α and/or EVT-401 (Fig. 5A). First, we compared P2X7R expression in RA SF stimulated with TNF-α for 24 hours to that in the vehicle-treated controls. The results showed that TNF-α stimulation increased P2X7R expression (Fig. 5B). On the basis of previous results, we selected drug concentrations of 10 and/or 100 μM for the experiment. A hallmark of SF is their enhanced secretion of inflammatory cytokines. To evaluate this, we treated SF derived from patients with RA and TNF-α, with or without EVT-401, for 24 hours and quantified the subsequent secretion of IL-6 and IL-8 using enzyme-linked immunosorbent assay (ELISA). The results revealed that 100 μM EVT-401 markedly reduced TNF-α–induced IL-6 secretion, whereas its effect on IL-8 secretion was minimal (Fig. 5C). In addition, EVT-401 attenuated the expression levels of tissue-destructive mediators such as matrix metalloproteinase 3 (MMP-3) and Dickkopf-1 (DKK-1) in TNF-α–stimulated fibroblasts (Fig. 5C).

To further evaluate the effects of EVT-401 on SF behavior, we investigated its influence on cell migration and invasion. In a monolayer wound-scratch assay, EVT-401 treatment at 10 μ0 significantly reduced the migratory capacity of RA SF compared to cells treated with TNF-α alone during 24 hours (Fig. 5, D and G). Similarly, EVT-401 also suppressed TNF-α–induced transwell migration (Fig. 5, E and H). Furthermore, EVT-401 treatment inhibited the invasive potential of RA SF through Matrigel-coated transwell membranes (Fig. 5, F and I), corroborating the migration inhibition findings. Together, these results demonstrate that the P2X7R antagonist effectively suppresses the production of proinflammatory cytokines and tissue-destructive mediators while reducing the migratory and invasive phenotypes of RA SF. These actions highlight its potential to mitigate inflammatory damage and protect against cartilage and bone destruction in RA.

### EVT-401 inhibited pathways associated with refractory RA in SF

**Fig. 6.**
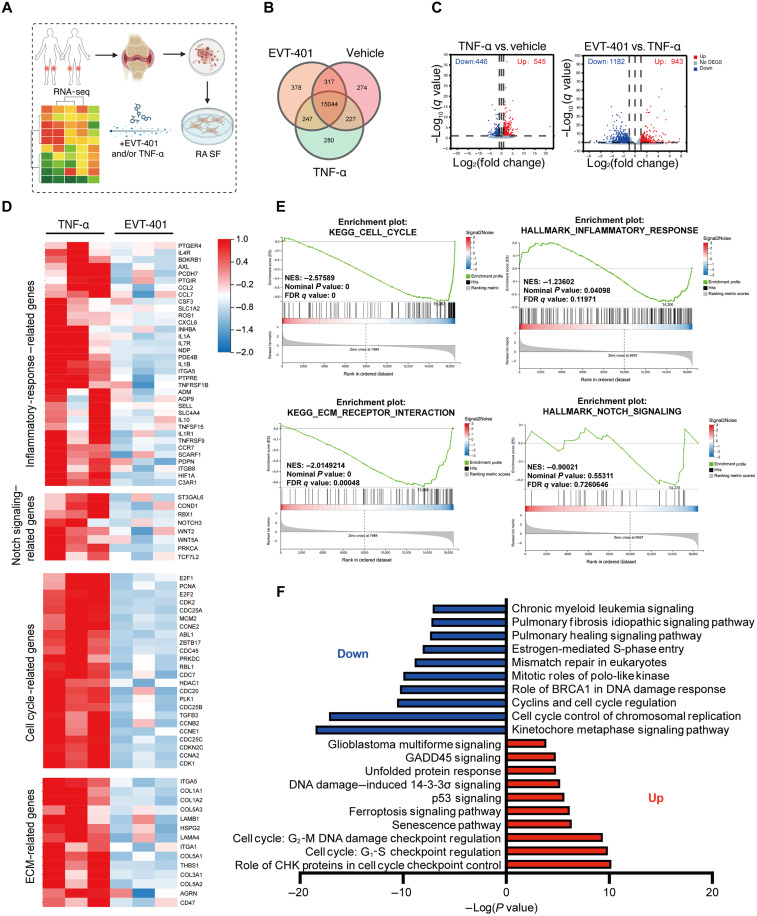
Transcriptome profile of EVT-401 group in RA. (**A**) SF were isolated and cultured from synovial tissue of patients with RA and treated with TNF-α (20 ng/ml) alone and TNF-α (20 ng/ml) plus 100 μM EVT-401 (*n* = 3 in each). After 24 hours, RNA-seq was performed on these SF samples. (**B**) Venn diagram showing the up-regulated and down-regulated genes in the TNF-α group compared to the EVT-401 and vehicle groups. (**C**) Volcano plot displaying DEGs with significant up-regulation or down-regulation [log_2_ (fold change) ≥ 1; q ≤ 0.05] across different groups. (**D**) Heatmap showing genes that are relevant to different pathway. (**E**) Enrichment plots of KEGG_cell cyle and inflammatory response gene set among the EVT-401 group identified by GSEA. GSEA showing cell cycle, inflammatory response, ECM receptor interaction, and NOTCH signaling enriched in the EVT-401–treated group compared with the TNF-α group. NES, normalized enrichment score. The *P* value is calculated through permutation tests. (**F**) Ingenuity Pathway Analysis (IPA) showing canonical pathways that were differentially up-regulated and down-regulated in the EVT-401 group compared to the TNF-α group (EVT-401 group: RA SF + TNF-α + EVT-401, TNF-α group: RA SF + TNF-α + 0.1% DMSO, and vehicle: RA SF + 0.1% DMSO). Created in BioRender. Shi, Y. (2026) https://biorender.com/rb2ok5q.

To elucidate the mechanisms by which P2X7R inhibition modulates SF function in RA, we performed RNA-seq on RA SF treated with TNF-α in the presence or absence of 100 μM EVT-401 for 24 hours (Fig. 6A). The experimental groups included resting RA SF (RA SF group), TNF-α–activated RA SF (TNF-α group), and TNF-α–activated fibroblasts cotreated with EVT-401 (EVT-401 group). As shown in the Venn diagram (Fig. 6B), the transcriptomic profiles of the EVT-401 group were markedly distinct from those of both the RA SF and TNF-α groups. Specifically, 943 genes were up-regulated, and 1182 genes were down-regulated in the EVT-401 group compared to the TNF-α group, with log_2_ (fold change) ≥ 1.0 and *q* ≤ 0.05 (Fig. 6C and data files S3 to S6). The heatmap (Fig. 6D) highlighted that EVT-401 significantly down-regulated the expression of genes involved in inflammatory responses, cell cycle regulation, the Notch signaling pathway, and ECM organization—pathways that are enriched in SF subsets associated with treatment-resistant RA. Gene set enrichment analysis (GSEA) confirmed significant down-regulation of pathways related to cell cycle progression, inflammatory responses, ECM interactions, and Notch signaling in EVT-401 group compared to the TNF-α group (Fig. 6E).

To further explore canonical pathways affected by EVT-401, we analyzed differentially expressed genes (DEGs) using Ingenuity Pathway Analysis (IPA). Among the pathways identified “cell cycle” was the most significantly modulated, followed by the “senescence pathway” and the “ferroptosis signaling pathway” (Fig. 6F). In summary, EVT-401 effectively suppressed pathogenic pathways associated with refractory RA in SF, with cell cycle regulation emerging as a central mechanism of its therapeutic efficacy as a P2X7R antagonist.

### In vivo validation of EVT-401 efficacy in an NHP arthritis model and a humanized cartilage–RA SF coimplantation model

To investigate the in vivo therapeutic efficacy of EVT-401, we initially used a collagen-induced arthritis (CIA) model in female Lewis rats. The rats received varying concentrations of EVT-401 or dexamethasone (DEX) via oral gavage (fig. S6A).

Arthritis severity was evaluated through longitudinal monitoring of body weight, paw volume, and clinical scores. Despite detectable intergroup variations in blood drug levels (fig. S6B), EVT-401 showed no significant therapeutic efficacy in this arthritis model (fig. S6, C to E), which was concordant with its previously reported weak antagonistic activity at the rat P2X7R (table S7).

**Fig. 7.**
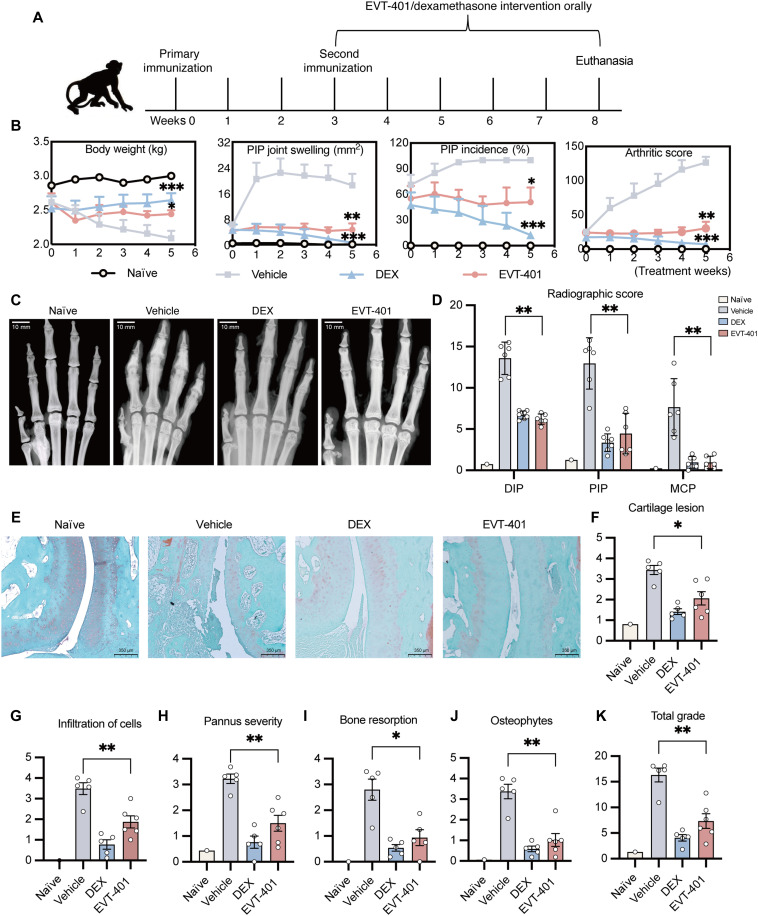
In vivo efficacy of EVT-401 in NHP arthritis. (**A**) Experimental timeline illustrating the immunization and treatment protocols. (**B**) Body weight, incidence of PIP arthritis, PIP joint swelling, and arthritis scores were assessed every 7 days across four groups: naïve control (*n* = 1), vehicle-treated CIA animals (*n* = 7), DEX-treated CIA animals (*n* = 7), and EVT-401–treated CIA animals (*n* = 8). Statistical analyses were performed using the Kruskal-Wallis test with Dunn’s post hoc correction. (**C** and **D**) Representative x-ray images of forepaws and statistical comparison of DIP, PIP, and MCP joint radiographic scores evaluated using established radiographic scoring criteria. Radiographic analyses included naïve control (*n* = 1), vehicle-treated CIA animals (*n* = 6), DEX-treated CIA animals (*n* = 7), and EVT-401–treated CIA animals (*n* = 6). Each data point represents the mean radiographic score from a single animal. Scale bars, 10 mm. (**E** to **K**) Representative Safranin O–stained histological sections of PIP joints from forelimb digits of cynomolgus monkeys in the indicated treatment groups. For quantitative histopathological analyses, naïve control (*n* = 1), vehicle-treated CIA animals (*n* = 5), DEX-treated CIA animals (*n* = 5), and EVT-401–treated CIA animals (*n* = 6) were included. Histopathological features, including total scores, cartilage lesions, osteophyte formation, bone erosion, inflammatory cell infiltration, and pannus severity, were evaluated on day 40. For panels (E) to (K), each data point represents the mean histological score of 16 PIP joints from a single animal. Scale bars, 350 μm. Comparisons between the EVT-401–treated and vehicle-treated groups were performed using the Mann-Whitney *U* test. Data are presented as mean ± SEM. *P < 0.05, **P < 0.01, and ***P < 0.001 versus vehicle group. Created in BioRender. Shi, Y. (2026) https://biorender.com/67w01r2.

To overcome the limitation of the murine disease models, we used a cynomolgus monkey CIA model for further investigation (Fig. 7A). The vehicle, DEX, and EVT-401 treatment groups are compared against a naïve control.

Our findings demonstrated that EVT-401 significantly reduced the clinical arthritis score, diminished swelling in proximal interphalangeal (PIP) joints, and decreased the incidence of PIP arthritis (Fig. 7B). Radiographic assessments of distal interphalangeal (DIP), PIP, and metacarpophalangeal (MCP) joints indicated that EVT-401 provided protective effects against bone erosion (Fig. 7, C and D). Histological evaluations were performed on PIP joint specimens to assess bone resorption and cartilage damage using Safranin O staining. PIP joints from the index, middle, ring, and little fingers of both forelimbs and hind limbs (16 PIP joints per animal) were included for analysis. Figure 7E shows representative histological sections of PIP joints from the forelimb digits. The results showed that EVT-401 markedly reduced cartilage destruction in the arthritis model. Histopathological scores for cynomolgus monkeys treated with EVT-401 at a dosage of 100 mg/kg were significantly lower than those of the vehicle control group (Fig. 7, F to K).

To assess systemic inflammatory responses during treatment, we monitored serum CRP levels in cynomolgus monkeys with CIA. As shown in fig. S7A, serum CRP levels remained consistently low in the naïve group but increased markedly in vehicle group. Treatment with either DEX or EVT-401 led to a progressive decline in CRP levels over time, with DEX inducing a more rapid reduction. Although intergroup differences did not reach statistical significance, a downward trend was observed in the EVT-401 group (fig. S7A). We next investigated the relationship between CRP levels and local joint inflammation. CRP levels positively correlated with arthritis scores (Spearman *r* = 0.32, *P* = 0.0005; fig. S7B) and joint swelling (*r* = 0.25, *P* = 0.0069; fig. S7C), supporting the close link between systemic inflammatory responses and local joint pathology. Furthermore, H&E staining of the heart, liver, spleen, lung, and kidney revealed no apparent pathological damage in the EVT-401–treated group (fig. S8).

**Fig. 8.**
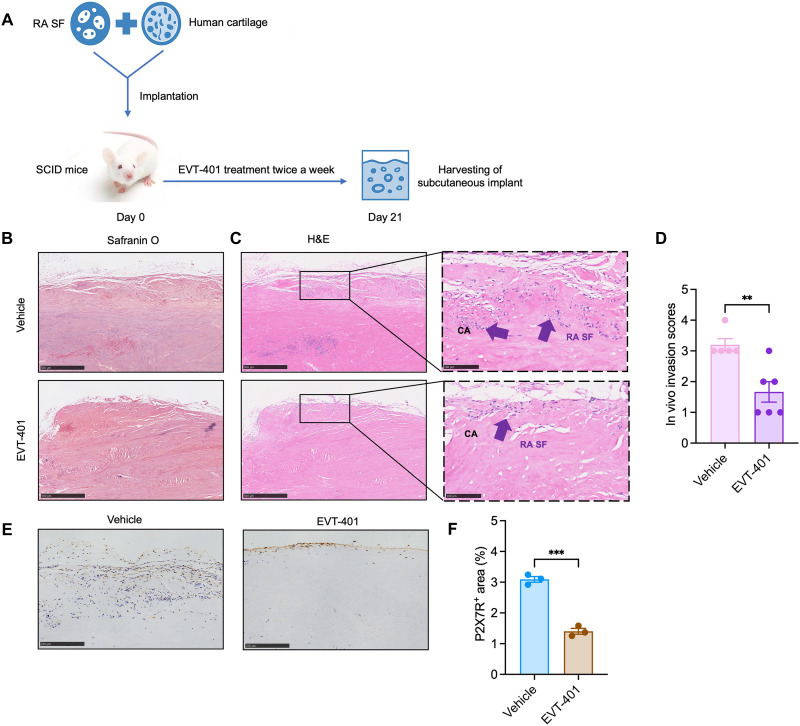
EVT-401 attenuates RA SF invasiveness in a humanized SCID mouse model. (**A**) Schematic diagram of the experimental design. RA SF and human cartilage were coimplanted subcutaneously into NOD-SCID mice (day 0). EVT-401 (100 μM) or vehicle (0.1% DMSO) was administered twice weekly via peri-implant subcutaneous injection. Implants were harvested on day 21 for histological analysis. (**B**) Representative histological sections of the implants stained with Safranin O (left panels; scale bar, 500 μm) and H&E (right panels; low magnification, scale bar, 500 μm; high magnification, scale bar, 100 μm). CA, cartilage; purple arrows indicate invading RA SF within cartilage. (**C**) Representative histological sections of the implants stained with H&E (low magnification, scale bar, 500 μm; high magnification, scale bar, 100 μm). Arrows indicate invading RA SF within cartilage. (**D**) Histological scoring of cartilage degradation based on H&E staining (*n* ≥ 5). (**E**) Representative IHC staining of hP2X7R in cartilage sections (*n* = 3; scale bars, 250 μm). (**F**) Quantification of hP2X7R-positive area within human cartilage implants. Data are shown as mean ± SEM. Statistical analyses were performed using two-tailed unpaired *t* test. **P < 0.01 and ***P < 0.001 versus vehicle group. Created in BioRender. Shi, Y. (2026) https://biorender.com/zji9vws.

While the NHP model provided proof-of-principle evidence for in vivo efficacy, it did not directly demonstrate the therapeutic effects of EVT-401 on human RA SF in vivo. To address this, we further used a humanized cartilage–RA SF coimplantation model in nonobese diabetic mice (NOD)–severe combined immunodeficient (SCID) mice (Fig. 8A). In this model, primary RA SF were coimplanted with human cartilage and treated with EVT-401 or vehicle. Histological analyses revealed that EVT-401 significantly inhibited the invasion of RA SF into the cartilage, as evidenced by Safranin O and H&E staining (Fig. 8, B to D). Immunohistochemical analysis showed that the expansion of hP2X7R-positive RA SF into cartilage was inhibited by EVT-401 treatment compared to vehicle-treated controls (Fig. 8E), supporting that EVT-401 acts on the human cartilage–RA SF coimplants to suppress hP2X7R-expressing RA SF expansion and invasion into cartilage. Quantification of hP2X7R-positive area confirmed this observation (Fig. 8F). Together with the NHP results, these findings demonstrate that EVT-401 exerts direct anti-invasive and cartilage-protective effects on human RA SF in vivo, supporting that it might become a potential therapeutic strategy for RA.

## DISCUSSION

Previous studies reported that P2X7R expression was up-regulated in peripheral blood mononuclear cells (PBMCs) of patients with RA compared to healthy controls (*28*, *36*). In addition, Caporali *et al.* (*37*) confirmed the presence of functional P2X7R in in vitro cultured SF from patients with RA, but the precise expression patterns and functional roles of P2X7R in RA SF subsets have not been fully elucidated. The rationale that P2X7R might be a causal factor of autoimmune arthritis is mainly supported by previous functional and genetic evidence. P2X7R-deficient mice exhibit reduced susceptibility and/or disease severity in the anticollagen antibody–induced arthritis model, providing genetic evidence for a contributory role of P2X7R in autoimmune arthritis (*38*, *39*). One study demonstrated that P2X7R signaling is essential for Th17 cell differentiation induced by type II collagen, and pretreatment with pharmacological antagonists of P2X7R (Suramin and A-438079) decreased murine CIA progression (*28*). Furthermore, P2X7R exacerbates RA progression by mediating serum amyloid A–dependent antiapoptotic signaling in neutrophils, thereby prolonging their survival and amplifying chronic inflammation (*30*). Notably, macrophages derived from PBMCs of patients with anti-citrullinated protein antibody–positive RA exhibit elevated IL-1β production via a P2X7R/NLR family pyrin domain containing 3 (NLRP3) inflammasome–dependent pathway (*29*). These findings highlight the multifaceted role of P2X7R in RA pathogenesis, yet its specific contribution to disease persistence and its value as a therapeutic target remain to be clarified.

Current RA treatments include conventional synthetic DMARDs (csDMARDs) such as methotrexate, biological agents targeting key immune pathways (e.g., TNF-α, CD20^+^ B cells, and IL-6R), and targeted synthetic DMARDs, including Janus kinase (JAK) inhibitors (*40*). Refractory RA is defined by resistance to csDMARDs and at least three targeted therapies spanning distinct immunological pathways (*4*). This phenotype is associated with pauci-immune synovial histology, where fibroblasts play a predominant pathogenic role. Zhang *et al.* (*20*) further identified the CTAP-F subtype, characterized by a high prevalence of fibroblasts, as the multidrug-resistant group within the R4RA cohort.

Although therapies targeting SF, such as ASP5094 targeting integrin α_9_ (*41*), have been explored, these approaches have often failed to demonstrate efficacy. Currently, drugs targeting SF remain under investigation. It has also been reported that the P2X7R plays a role in cell proliferation (*42*), cytokine release (*43*), and cell death (*44*). Bianchi *et al.* (*45*) demonstrated that P2X7R modulated myeloid-derived suppressor cells’ immunosuppressive functions. However, there is limited evidence regarding the impact of P2X7R on SF.

In this study, we identified marked overexpression of P2X7R in RA synovium and SF, with expression levels correlating with synovitis severity and systemic inflammation. Single-cell transcriptomic analysis mapped P2X7R predominantly to PRG4^+^CLIC5^+^ (F-0), PRG4^+^ (F-1), and RSPO3^+^ (F-8) lining subsets and to DKK3^+^ (F-4) and NOTCH3^+^ (F-7) sublining subsets. P2X7R expression was particularly enriched in the CTAP-F subtype, a multidrug-resistant stromal phenotype associated with poor clinical response. Functional enrichment analyses revealed activation of ECM organization, Notch signaling, and cell adhesion pathways in P2X7R^+^ SF, supporting a role of P2X7R in stromal-driven refractory RA. Notably, up-regulation of P2X7R and its association with disease activity and synovitis are insufficient to establish its causal role in development of refractory RA. Because of lack of animal models mimicking refractory RA, we are unable to prove that P2X7R is one of the causal factors of refractory RA animal models either. Future studies on proper animal models are in need to reveal the contribution of P2X7R in refractory RA.

P2X7R was reported to promote proinflammatory factor release, such as IL-6 from human fibroblasts (*46*). Besides detected overexpression in RA SF, direct evidence for substantial P2X7R expression in fibroblast-rich normal tissues under homeostatic conditions remains limited. While low-level expression has been reported in dermal fibroblasts from healthy skin (*47*), and its up-regulation has been demonstrated in intestinal fibroblasts under inflammatory conditions (*48*), few studies to date have demonstrated high basal expression of P2X7R in fibroblasts from noninflamed lymphoid tissues. In lung tissues, normal alveolar epithelium, vascular endothelial cells, and a small number of alveolar macrophages exhibit low expression of P2X7R. In bleomycin or silicosis models, myofibroblasts rapidly show elevated P2X7R levels, associated with collagen deposition and MMP-9/TIMP-1 imbalance (*49*). In kidney, P2X7R is minimally expressed in normal renal parenchyma. During days 3 to 7 after ureteral obstruction, P2X7R was significantly up-regulated in renal mesenchymal fibroblasts and myofibroblasts, driving the production of IL-1L-rtransforming growth factor–β (TGF-βG), and collagen types I, III, and IV (*50*). In heart, P2X7R shows low expression in cardiac fibroblasts but rapidly increases in response to stress, TGF-β1, or angiotensin II stimulation. This up-regulation activates fibroblast proliferation and cardiac fibrosis. Blocking P2X7R effectively inhibits these pathological responses, suggesting it as a potential therapeutic target for cardiac fibrosis (*51*). Similar “inflammation–fibroblast activation–P2X7R up-regulation” phenomena also exist in other tissues such as skin (chronic wounds) and liver (fibrosis) (*49*). Collectively, these data support the inference that P2X7R up-regulation might be a hallmark of disease-associated stromal activation, consistent with our findings of elevated P2X7R expression in RA synovium compared with relatively lower levels in OA controls, which are under a milder inflammation condition. Because blockade of P2X7R might decrease pathogenic autoimmunity or inflammation and P2X7R expressions might be elevated in stromal fibroblasts under autoimmune or inflammatory conditions, P2X7R inhibition might become an potential target therapy for a variety of autoimmune or inflammatory conditions, not only limited to RA.

Mechanistically, our data suggest that P2X7R may contribute to SF activation through Notch signaling and cell cycle regulation. P2X7R activation induces Ca^2+^ influx, which is known to influence sheddase activity (e.g., ADAM) and NICD generation to activate Notch signaling (*52*). In our dataset, P2X7R^+^ SF subtypes displayed up-regulation of Notch and E2F target genes, implicating this axis in promoting proliferation and sustaining a proinflammatory phenotype. Our further analyses integrated expression, modules, and networks to propose a specific P2X7R → Ca^2+^ → Notch/CDK mechanism. First, Module-1 activity correlates with P2RX7 at single-cell resolution (fig. S3), linking P2X7R to a transcriptional program enriched for Notch and cell-cycle signatures. Second, Module-1’s core network contains Notch-processing/tuning hubs—ADAM10, TIMP3, and LFNG—together with MAPK7 [a Ca^2+^-responsive MAPK supporting activator protein-1 (AP-1)/nuclear factor of activated T cells (NFAT)] (fig. S3A). These features suggest that Ca^2+^influx downstream of P2X7R might modulate sheddase activity and LFNG regulated Notch receptor glycosylation to promote NICD generation and then engage CDK-related programs. We therefore provide a possible route by which P2X7R couples to Notch and cell-cycle control. Our further in vitro experiment proved that EVT-401 inhibited RA SF overproliferation by arresting cell cycle progression at the S phase via Cyclin E1/Cdk2 suppression (Fig. 4G). These effects align with the known role of Notch activation in driving synovial expansion and multidrug resistance (*32*).

Previous P2X7R-targeting clinical trials in RA, such as AZD9056 and CE-224,535, failed to achieve significant efficacy despite promising preclinical data (*53*). These failures may be attributed to inadequate target engagement arising from suboptimal selectivity for hP2X7R, lack of patient stratification—unselected RA populations were enrolled rather than those enriched with P2X7R-high SF subtypes such as CTAP-F—and insufficient use of pharmacodynamic biomarkers to confirm on-target activity.

Notably, some early antagonists showed higher affinity for rodent than for hP2X7R, raising translational concerns. Michel *et al.* (*54*–*56*) identified protein regions and key amino acid residues that contribute to the divergent antagonist sensitivity between human and rat P2X7R, thereby providing a structural basis for species-dependent drug response and underscoring the limitations of rodent models for preclinical evaluation. These findings are directly pertinent to our study, as they underscore the translational limitations of rodent models and highlight the necessity of testing EVT-401 in human cells and humanized in vivo disease models. NHPs, whose P2X7R is more homologous to that of humans, might also be more suitable for P2X7R antagonist efficacy testing than rodent models.

Building on these lessons, EVT-401 was designed to achieve high selectivity for hP2X7R and tested in an NHP model to enhance translational relevance. Nevertheless, a key limitation of our study is the absence of direct head-to-head comparisons between EVT-401 and earlier antagonists. Although EVT-401 demonstrated potent binding affinity, strong human selectivity, and efficacy in the NHP model, it remains unclear whether these properties confer clear advantages over prior compounds. Future studies should therefore include comprehensive comparative analyses encompassing target affinity, binding sites, human receptor selectivity, and efficacy across multiple animal models. These systematic evaluations will be critical not only to clarify the therapeutic potential of EVT-401 but also to inform broader drug development strategies targeting hP2X7R.

To guide future clinical trials, raising a translational roadmap might be helpful. First, P2X7R expression profiling from synovial biopsies should be performed to stratify patients with RA for enrollment. Early-phase trials should adopt dose-escalation designs to define the optimal therapeutic window while minimizing adverse effects and incorporate early pharmacodynamic endpoints (e.g., changes in SF activation markers or serum inflammatory mediators) to confirm target engagement. Interactions between EVT-401 and existing RA therapies (e.g., TNF inhibitors and JAK inhibitors) should also be evaluated early, as synergistic or antagonistic effects may substantially influence clinical outcomes. Mechanistically, P2X7R activation drives upstream inflammatory cascades (*57*)—including NLRP3 inflammasome assembly, IL-1β/IL-18 maturation, and nuclear factor κB/MAPK activation—which converge on cytokines (IL-1β, IL-6, and TNF-α) targeted by current biologics and JAK inhibitors. This places P2X7R inhibition upstream of JAK–signal transducers and activators of transcription and TNF/IL-1 pathways, suggesting additive or synergistic potential when combined with these agents by simultaneously reducing cytokine production and blocking downstream signaling. While direct pharmacologic antagonism is unlikely, overlapping immune suppression could increase infection risk, warranting careful safety monitoring in combination studies. Given the potential for P2X7R blockade to modulate immune surveillance, long-term safety assessment should include monitoring for infections and tumor development.

There are several limitations in this study. First, the relatively small synovial sample size limits the generalizability of our findings for both RA and OA cohorts. While we observed elevated P2X7R expression in patients with CTAP-F RA, further clinical trials and datasets are needed to validate its role as a biomarker for patient selection and therapeutic response prediction. Second, the temporal dynamics of P2X7R expression during RA progression or treatment remain unclear, necessitating longitudinal tissue biopsy studies. Third, the mechanistic interplay between P2X7R, Notch signaling, and other pathways warrants deeper investigation using regulatory network modeling and functional validation. Fourth, an important limitation of our work is the inability to dissect the contributions of P2X7R inhibition in stromal cells and immune cells, respectively, to the overall therapeutic effects of EVT-401. Because EVT-401 showed no obvious in vivo efficacy in rodent P2X7R inhibition, comparison of treatment efficacy of two bone marrow–chimeric rodent autoimmune arthritis models with selective expression of the hP2X7R on hematopoietic cells or stromal cells, respectively, would be helpful to provide concrete evidence for the significance of direct hP2X7R inhibition on stromal cells by EVT-401. This study is mechanistically critical but cannot currently be addressed in vivo for the hP2X7R knock-in or transgenic mice are not available. Future studies on hP2X7R knock-in mice and bone marrow–chimeric models will be required to clarify these contributions. Fifth, although EVT-401’s functional selectivity to hP2X7R over its related receptors has been shown by in vitro experiments in this study, similar to many other small molecular antagonists, the off-target effects of EVT-401 might also exist. It might bind other targets in vivo, which might contribute to its therapeutic effects or even induce many other effects. A deep and broad target screening should be performed in future studies to further elucidate this point. Because the possible off-target effects of EVT-401 cannot be absolutely excluded, therapeutic effects in vitro and in vivo are still unable to justify P2X7R as a causal factor of autoimmune arthritis. Last, while the NHP model provides valuable in vivo insights, species differences in disease course and tissue remodeling still highlight the need for cautious extrapolation to human RA. In our NHP CIA model, EVT-401 significantly alleviated arthritis symptoms, particularly ameliorating bone destruction and synovial pathology. Imaging-based assessment of joint involvement and structural damage is widely used across inflammatory arthritides, including gouty arthritis (*58*). The higher genetic and pharmacological similarity between humans and cynomolgus macaques compared to rodent models supports the translational potential of these findings. Nonetheless, NHP arthritis still differs from human RA in the extent of synovial fibrosis, immune cell infiltration patterns, and chronicity (*59*–*62*), which could influence efficacy assessment—potentially favoring agents with pronounced effects in acute inflammatory phases over those targeting chronic remodeling. Although the NHP CIA model offers valuable support of in vivo target engagement and therapeutic efficacy, these results should be interpreted as proof of principle rather than direct predictors of human clinical outcomes. The EVT-401 concentrations used for in vitro RASF experiments are higher than its plasma concentrations in the in vivo NHP experiment. This suggests that the in vitro SF experiments might not completely mimic the actual drug levels in vivo and might induce different effects or mechanisms compared with the in vivo treatment. Future studies in more humanized arthritis models and longitudinal patient cohorts with proper biopsy tests during the treatment will be essential for validating EVT-401’s efficacy and revealing its exact mechanism in vivo.

In conclusion, our findings identify P2X7R overexpression in SF as a key feature of refractory RA, particularly in the CTAP-F subtype. EVT-401, a selective hP2X7R antagonist, effectively suppressed pathogenic SF phenotypes in vitro and ameliorated arthritis in a primate model. Inhibiting P2X7R in SF might represent a potential therapeutic approach for refractory RA, warranting further evaluation in biomarker-stratified clinical trials.

## MATERIALS AND METHODS

### Patients and synovial tissues

Synovial tissues from patients with RA and OA were collected during synovectomy or joint replacement surgeries, following the 2010 American College of Rheumatology/EULAR criteria (*63*). All patients with RA included in the study exhibited poor responses to drug treatments, resulting in joint dysfunction that required surgical intervention. Written informed consent was obtained from all participants, and the study received ethical approval from the Ethics Committee of Peking University People’s Hospital (approval no. 2021PHB257-001 and 2022PHB112-001).

### IHC of synovial tissues

Synovial tissues were fixed in 7.5% buffered paraformaldehyde and subsequently embedded in paraffin, following standard histological protocols. Immunohistochemical analysis was conducted to detect P2X7R expression using a polyclonal rabbit anti-P2X7R antibody (#28207-1-AP). Afterward, sections were incubated with a biotinylated goat anti-rabbit secondary antibody. This was followed by incubation with a biotinylated goat anti-rabbit secondary antibody. Sections were then treated with VECTASTAIN Elite reagent, and staining was visualized using 3,3-diaminobenzidine (Vector, Burlingame, CA, US). Hematoxylin (Merck, Darmstadt, Germany) was used for counterstaining. Microscopic images were captured with Olympus Digital Camera DP73 using the cellSens Dimension software. P2X7R expression levels were assessed independently by two evaluators using a semiquantitative scoring system (RA: *n* = 22; OA: *n* = 10).

### Cell culture and treatment

RA SF were isolated from synovial tissue samples obtained from patients with RA through enzymatic digestion with type I collagenase (1 mg/ml; Sigma-Aldrich). The isolated cells were cultured in Dulbecco’s modified Eagle’s medium (DMEM; Gibco) supplemented with 10% fetal bovine serum (FBS; Gibco) and 1% penicillin/streptomycin solution (Gibco). Cultures were maintained under normoxic conditions and used for experiments at passages 4 to 7 to ensure cellular homogeneity and functional consistency. For experimental protocols, EVT-401 was first dissolved in dimethyl sulfoxide (DMSO) and then diluted with culture medium, with working concentrations ranging from 10 to 200 μM.

### RN-0278885 saturation binding assay

All binding studies were conducted using human embryonic kidney (HEK) 293 cells transfected with linear cDNA encoding hP2X7. Stable HEK 293 A6 clonal cells expressing hP2X7 protein were cultured on 40 15-cm tissue culture–treated plates and harvested at 85 to 100% confluency. Cells were washed once with phosphate-buffered saline (PBS) and detached using cold Versene. The collected cells were centrifuged at 485*g* for 4.5 min (benchtop Sorvall Legend RT). After discarding the supernatant, frozen cell pellets were resuspended in homogenization buffer [10 mM Hepes, 1 mM EDTA (pH 7.4), and 0.5% protease inhibitor cocktail] and thawed on ice.

For homogenization, the cell suspension was processed using a Polytron homogenizer at setting 6 for 15 s, rested on ice for 30 s, and homogenized again for 15 s. The homogenate was centrifuged at 1000*g* for 10 min at 4°C to remove debris, followed by a second centrifugation at 2600*g* for 5 min at 4°C. The supernatant was subjected to ultracentrifugation at 18,500*g* for 30 min at 4°C (Sorvall Discovery 90 ultracentrifuge). The resulting pellet was resuspended in 4 ml of homogenization buffer and homogenized for 15 s at setting 6.

Protein concentration was measured using the Advanced Protein Assay reagent. Membrane preparations (0.041 mg of protein per well) were incubated in triplicate in a 96-well filter plate with increasing concentrations of [^3^H]-RN-0278885 (0.001 to 31 nM) for 6 hours at room temperature, with or without 1 μM nonradioactive RN-0278885. Bound and free ligands were separated by vacuum filtration through GF/C glass fiber filters (pore size: 0.65 μm) and washed with ice-cold assay buffer containing α_1_-acid glycoprotein (0.5 mg/ml).

The filters were dried and transferred to scintillation vials. Scintillation fluid (3 ml) was added, and samples were incubated overnight at room temperature. Bound ligand was quantified using a Beckman LS 6500 scintillation counter.

### Schild experiments

The hP2X7 membrane preparation (0.024 mg) was incubated with increasing concentrations of [^3^H]-RN-0278885 (0.18 nM to 112 μM) in the presence of EVT-401 at various concentrations (0, 10, 31.6, 100, and 316 nM) in assay buffer. The assay components were allowed to equilibrate in a 96-well filter plate for more than 36 hours at room temperature. Nonspecific binding was defined as the binding observed in the presence of either 1 μM nonradioactive RN-0278885 or 1 μM EVT-401.

After incubation, bound ligand was separated from free ligand by filtration and washing, as described previously. The filters were dried and lined with a MultiScreen liner before adding 200 μl of scintillation fluid to each well. Radioactivity was measured using a Wallac MicroBeta Jet 1450 liquid scintillation counter. For each concentration of [^3^H]-RN-0278885, in the absence or presence of EVT-401, duplicate wells were assayed.

### Human and rat P2X7 IC_50_ determinations

The function of P2X7R can be assessed by monitoring calcium influx into cells. The astrocytoma cell line 1321N1, which lacks an endogenous calcium response to P2X7 agonists, provides an ideal background for expressing P2X7 and evaluating its function. Human and rat P2X7–expressing 1321N1 cells were prepared and stored in single-use frozen aliquots.

Twenty-four hours before the assay, hP2X7-expressing cells were plated at a density of 50,000 cells per well, and rat P2X7–expressing cells were plated at 40,000 cells per well in a 96-well poly-l-lysine–coated Packard plate. The culture medium consisted of DMEM (without phenol red) supplemented with 10% FBS, penicillin (100 U/ml), streptomycin (100 μg/ml), 2 mM GlutaMAX, and G-418 (500 μg/ml). Cells were incubated at 37°C in a humidified atmosphere containing 5% CO_2_. Before the assay, the medium was removed and replaced with 100 μl of dye loading solution per well. This solution comprised 10 ml of Hanks’ balanced salt solution (HBSS) with 20 mM Hepes, supplemented with 100 μl of 250 mM probenecid, 500 μl of signal enhancer, and 5 μl of dye from the BD Calcium Kit. Cells were incubated with this dye solution for 60 min at 37°C and 5% CO_2_, followed by additional 10-min incubation at room temperature. EVT-401 was added to each well by dispensing 100 μl of a 2.22× stock solution to achieve the desired final concentration. Cells were pretreated with EVT-401 for 30 min at room temperature. Subsequently, 22.2 μl of a 10× stock solution of the agonist was added to each well, and fluorescence was recorded over a 90-s interval using the FLIPR Tetra system. The agonist BzATP was used at the EC_80_ concentration for each receptor type.

The maximum and minimum relative fluorescence units were calculated for each well during the first 90 s after agonist addition. Background fluorescence, determined from wells without agonist, was subtracted, and values were normalized to the maximum possible effect observed in wells treated with the agonist alone (without EVT-401). Data were expressed as the observed effect divided by the maximum possible effect (*E*/*E*_max_). The IC_50_ values were calculated using a four-parameter logistic curve-fitting algorithm in GraphPad Prism (GraphPad Software Inc., San Diego, CA).

### Selectivity against human P2X1, P2X2, P2X3, and P2X4

1321N1 cells expressing human P2X1, P2X2, P2X3, and P2X4 receptors were stored in single-use frozen aliquots. Twenty-four hours before the assay, the cells were plated under the following conditions:

1) P2X1: 60,000 cells per well in DMEM (without phenol red), supplemented with 10% FBS, penicillin (100 U/ml), streptomycin (100 μg/ml), 2 mM GlutaMAX, and G-418 (500 μg/ml).

2) P2X2: 75,000 cells per well in DMEM (without phenol red), supplemented with 10% FBS, 2 mM GlutaMAX, and hygromycin (200 μg/ml).

3) P2X3: 75,000 cells per well in DMEM (without phenol red), supplemented with 10% FBS, 2 mM GlutaMAX, and hygromycin (200 μg/ml).

4) P2X4: 100,000 cells per well in DMEM (without phenol red), supplemented with 10% FBS, 2 mM GlutaMAX, and hygromycin (200 μg/ml).

Before the assay, cells were loaded with a calcium-sensitive dye as described previously, with one modification: For P2X2 cells, the buffer was supplemented with 5 mM calcium instead of the standard 1.3 mM in HBSS. EVT-401 and BzATP were added as described previously. ATP was used as the agonist at the EC_80_ concentration specific to each receptor type.

### Molecular modeling

To assess the binding affinities and interaction mechanisms between the drug candidate EVT-401 and its target P2X7R, we used the Prepare Protein and Minimization protocols in Discovery Studio 2020 (BIOVIA, Dassault Systèmes, San Diego, United States). The three-dimensional (3D) structures of human and rat P2X7R proteins were retrieved from the Protein Data Bank using the codes 5U1U and 6U9W, respectively (accessible at www.rcsb.org/pdb/home/home.do). To identify the key interaction features of the P2X7R-EVT-401 interface, we applied the pharmacophore generation protocol from Drug Target Space (EGR Health, Beijing, China) using an artificial intelligence–based algorithm.

### CCK-8 assay

The viability of RA SF was assessed using a CCK-8 assay following standard protocols (*64*). RA SF were seeded in 96-well plates at a density of 1 × 10^3^ cells per well in 100 μl of culture medium, with five replicates for each condition. Cells were treated for varying durations (24, 48, and 72 hours). Following treatment, 10 μl of CCK-8 reagent (ZETA LIFE, China) was added to each well, and the plates were incubated for 2 hours at 37°C in the dark. After gentle shaking for 20 min at room temperature, the optical density at 450 nm (OD_450nm_) was measured using a microplate reader (Synergy).

### Flow cytometry

RA SF were seeded in six-well plates and cultured in DMEM supplemented with 10% FBS for 24 hours. For P2X7R determination, single cells were resuspended in PBS containing 1% P2X7R antibody (R&D). The samples were incubated for 30 min in the dark, washed with PBS, and resuspended in 200 μl of PBS. More than 10,000 cells were analyzed using a flow cytometer with the Alexa Fluor 488 detection channel.

RA SF were seeded in six-well plates and starved in DMEM supplemented with 1% FBS for 24 hours before being treated with EVT-401 for an additional 24 hours. For cell cycle analysis, the cells were fixed and permeabilized and then stained with propidium iodide (PI) in permeabilization solution for 30 min at 37°C. Flow cytometry was used to assess cell cycle distribution.

Apoptosis in RA SF was evaluated using the annexin V–7-AAD apoptosis assay kit (BioGems, China). Cells were stained with 5 μl of annexin V–fluorescein isothiocyanate and 5 μl 7-AAD in 200 μl of staining solution for 30 min. The percentage of apoptotic cells was analyzed using a FACSCalibur flow cytometer (BD Biosciences).

### Western blot

Immunoblotting was conducted using whole-cell lysates prepared in radioimmunoprecipitation assay buffer supplemented with a protease inhibitor and phosphatase inhibitor cocktail (Roche) following standard protocols (*65*). The lysates were incubated on ice for 30 min, and protein concentrations were determined using a bicinchoninic acid protein assay kit (Thermo Fisher Scientific). Thirty micrograms of protein per sample were loaded onto SDS–polyacrylamide gel electrophoresis gels for electrophoresis. After separation, proteins were transferred onto polyvinylidene difluoride membranes (Bio-Rad). The membranes were blocked with 5% skim milk for 1.5 hours and incubated overnight at 4°C with primary antibodies. Following incubation, the membranes were washed and treated with horseradish peroxidase (HRP)–conjugated secondary antibodies. Protein signals were visualized using an enhanced chemiluminescence system (Bio-Rad). Primary antibodies specific to the following proteins were purchased from Cell Signaling Technology: P2X7R (catalog no. 13809, dilution ratio: 1:1000), Cdk2 (catalog no. 18048, dilution ratio: 1:1000), cyclin E1 (catalog no. 20908, dilution ratio: 1:1000), p-Rb (catalog no. 8516, dilution ratio: 1:1000), Rb (catalog no. 9313, dilution ratio: 1:1000), and β-actin (catalog no. 4970, dilution ratio: 1:5000). β-Actin was selected as the internal reference control. The secondary antibody is the Goat Anti-Rabbit IgG H&L (HRP) from Cell Signaling Technology, with the catalog number 7074 and a dilution ratio of 1:5000.

### ELISA

Cell culture supernatants were thawed, and the levels of IL-6, IL-8, MMP-3, and DKK-1 were quantified using ELISA kits (LiankeBio, China). The assays were performed following the manufacturer’s instructions.

### Wound healing assay

RA SF were seeded in six-well plates, and wound lines were created by scratching the cellular monolayer with sterile 10-μl pipette tips. After 24 hours of incubation with EVT-401 and/or TNF-α, cell migration was quantified by counting the cells that had moved beyond a predefined reference line. Images were captured using an Olympus CKX31 microscope (Olympus Corporation, Tokyo, Japan).

### Transwell assays for vertical migration and invasion

The effect of EVT-401 on the vertical migration and invasion abilities of primary RA SF was evaluated using transwell assays. Primary RA SF (5 × 10^4^ cells) were seeded into 24-well plates and treated with EVT-401, with or without TNF-α (20 ng/ml), for 24 hours. For the migration assay, a transwell chamber with an 8-μm pore membrane (Corning, USA) was used. A total of 5 × 10^4^ cells was resuspended in serum-free medium and placed in the upper chamber, while the lower chamber was filled with complete medium. After a 24-hour incubation, cells remaining in the upper chamber were gently removed with a cotton swab. Migrated cells on the lower surface of the membrane were fixed with 4% paraformaldehyde and stained with 0.1% crystal violet. For the invasion assay, the upper chamber membrane was precoated with Matrigel (BD Biosciences). Cells were seeded in the same manner as the migration assay and allowed to invade for 24 hours. Cell counts were performed under a microscope at ×100 magnification. Data were expressed as the mean number of migrated or invaded cells, averaged from five randomly selected fields.

### RNA-seq analysis method

First, gene expression levels were calculated using RSEM (v1.3.1). A heatmap was generated with pheatmap (v1.0.8) based on the gene expression differences across different samples. Differential expression analysis was performed using DESeq2 (v1.4.5), DEGseq, or PoissonDis, with *q* ≤ 0.05 [or false discovery rate (FDR) ≤ 0.001]. Pathway and network analysis were conducted using QIAGEN’s IPA tool (www.qiagen.com/ingenuity).

### Single-cell RNA-seq data collected and further analysis

We obtained single-cell RNA-seq expression matrices, along with associated genotype and clinical data, from the Arthritis and Autoimmune and Related Diseases Knowledge Portal (https://arkportal.synapse.org/) under Synapse ID syn52297840, as generated by Zhang *et al.* (*20*). The single-cell data were derived from 82 biopsies collected from 79 donors (70 with RA, 9 with OA, and 3 repeated RA donors) and were aligned to the GRCh38 reference genome. Raw count matrices for all cells (*n* = 314,011) and reference data were imported into R (v4.4.0).

A subset of fibroblast-specific count matrices (*n* = 79,555) was curated, and a Seurat object was created using Seurat (v4.4.0) (*66*). Cell quality control was performed by excluding the following: (i) cells with >10 to 20% mitochondrial reads (indicating apoptosis or damage); (ii) cells with nCount_RNA < 500 (empty droplets) or > 50,000 (potential doublets); and (iii) cells with nFeature_RNA < 200 or > 6000 genes. Ribosomal gene percentages were assessed but not used as exclusion criteria.

The data were normalized, the top 3000 most variable genes were identified, and the data were scaled before performing principal components analysis (PCA; with parameter npc = 30) for dimensionality reduction. To address batch effects in multisample datasets, we applied Harmony integration (*67*), with key parameters using group.by.vars=orig.ident to remove its effect on the data.

Next, nearest neighbor analysis was performed on the Harmony-corrected data, followed by clustering with a resolution parameter of 0.5. Last, the data were projected into 2D using t-distributed stochastic neighbor embedding (t-SNE) and uniform manifold approximation and projection (UMAP) for visualization, based on the first 20 principal components corrected by Harmony.

To compare the expression of specific genes within CTAP groups and fibroblast subpopulations, we generated pseudobulk count matrices. First, RA single-cell RNA-seq expression counts were aggregated by sample and cluster number. Genes with expression counts below 30 were filtered out, followed by log_2_

counts per million (CPM) transformation. In addition, genes related to mitochondrial, ribosomal, and microRNA functions, as well as *MALAT1*, were excluded. Variable genes were identified using the FindVariableGenes function in Seurat and further filtered on the basis of gene.mean>3 and gene.dispersion>5. Next, a linear model matrix was constructed, incorporating grouping factors (e.g., group for cell groupings and donor for donor origin) and total expression level (cell_umi). The model formula was as follows

mm=model.matrix (∼0+group+donor+cell_umi)

DEGs were analyzed individually using the target gene set, and the results were combined into a complete data frame. DEGs were filtered on the basis of an adjusted *P* value threshold (Bonferroni correction) and a log_2_ fold change greater than 2.

The F-0 and F-1 subclusters of fibroblasts were subsetted and analyzed using Seurat v3 (*68*) in R. While most steps used default arguments, the FindVariableFeatures function required specific parameters (selection.method = “vst,” nfeatures = 5000). To detect and compare gene coexpression modules, we used the R package single-cell WGCNA (scWGCNA) (*69*) with the previously generated Seurat fibroblast subset object. Pseudocells were calculated with dims = 1:25, followed by running run.scWGCNA with default parameters. The *P2RX7* gene was identified within Module 1 (module black). Genes associated with module 1 were subsequently enriched using the Metascape. We then quantified a Module 1 gene signature score per cell using Seurat’s AddModuleScore, passing the Module 1 gene list; this function returns a control-adjusted average expression—the mean expression of Module 1 genes minus the mean of matched control genes—storing the score in the Seurat metadata for downstream comparisons. In addition, we derived a Module 1 eigengene from log-normalized data by genewise scaling, performing PCA, and taking PC1 as the eigengene (its sign aligned to P2RX7 and then *z* scored); association with P2RX7 was assessed using Pearson correlation and ordinary least-squares regression. To place Module 1 in pathway and network context, we ran overrepresentation analysis with clusterProfiler using MSigDB gene sets and computed within-module connectivity with WGCNA; hubs were defined as the top 10% by connectivity and mapped to curated NOTCH, AP-1/NFAT, and E2F/forkhead box protein M1 (FOXM1)–CDK axes (including ADAM10/17, TIMP3, and LFNG).

### Arthritis models and therapeutic evaluation

All experimental protocols and procedures were approved by the Animal Ethics Committee and conducted in strict compliance with relevant regulatory standards (PL13-0038A, PL13-0170, and 2024-461).

In accordance with previous studies (*70*), in the rat CIA experiments, female Lewis rats (6 to 8 weeks; *n* = 30) were immunized at the base of the tail with bovine type II collagen emulsified in an equal volume of complete Freund’s adjuvant in a total volume of 200 μl (final concentration: 1 mg/ml). A booster injection with the same amount of collagen in incomplete Freund’s adjuvant was administered on day 7. The rat CIA induction success rate was 100%. On day 7 after the first immunization, rats were randomized into five treatment groups (*n* = 6 per group) according to body weight using the randomization function of BioBook to ensure comparable mean body weights across groups and minimize intergroup bias. Treatments included vehicle (control), DEX (0.1 mg/kg), or EVT-401 at low (0.05 mg/ml), medium (0.5 mg/ml), or high (5 mg/ml) oral doses once daily. After the second immunization, arthritis severity was assessed twice a week by two independent assessors blinded to group allocation using a standardized clinical scoring system (0 to 4 per paw; maximum of 16 per animal). On the 28th day of the experiment, all animals were euthanized, marking the end of the experiment. All results are expressed as mean ± SEM. Differences between groups were analyzed using one-way analysis of variance (ANOVA), followed by Dunnett’s post hoc multiple comparisons. *P* < 0.05 was considered statistically significant. All statistical analyses were performed on the raw data.

In the NHP model (*71*), cynomolgus macaques (*Macaca fascicularis*; 3 to 4 years old) were anesthetized on day 0 and immunized intradermally with bovine type II collagen emulsion (final concentration: 2 mg/ml) at 20 sites across the back and the base of the tail. A booster injection was administered on day 21 using the same procedure. By day 42, the arthritis induction success rate in this model reached 100%. Arthritis severity was assessed beginning on days 0 and 21 and then weekly until the end of the experiment. The clinical arthritis index was graded on a 0-to-3 scale (0 = normal; 1 = mild arthritis with subtle but definite changes; 2 = moderate swelling; 3 = severe arthritis with substantial swelling and/or joint deformity) (*72*). For each paw, 15 joints were scored: 5 MCP, 4 PIP, 5 DIP, and 1 wrist or ankle joint, together with the four knee or elbow joints. The maximum cumulative score per animal was 192. Assessments were performed by two independent observers blinded to treatment allocation. Collagen-immunized animals were sequentially allocated to treatment groups once their arthritis score reached ≥5% of the maximum. Treatments included daily oral administration of EVT-401 (100 mg/kg) or DEX (10 mg/kg), in comparison with vehicle and control groups.

For radiographic evaluation, animals were anesthetized before radiographic examinations. DIP, PIP, and MCP joints were assessed using a 0-to-4 grading system. A score of 0 indicated normal joint architecture. A score of 1 indicated minor deformities in the articular cartilage layer and/or subchondral bone regions. A score of 2 indicated severe deformities in the articular cartilage layer and subchondral bone regions, accompanied by a small amount of osteophyte formation at periosteal surfaces; the joint cavity appeared blurred but remained identifiable. A score of 3 represented more advanced changes of those observed in grade 2, with extensive osteophyte formation at periosteal surfaces and an indistinguishable or invisible joint cavity. A score of 4 indicated further progression of grade 3 changes, characterized by complete loss of the joint cavity, sclerotic or ankylosing bone changes, and marked joint deformity. Each data point in the radiographic analysis represents the mean score across joints from a single animal.

After euthanasia, joint tissues were harvested for immunohistochemical analysis. Bone sections were decalcified (15% EDTA) and dehydrated before being embedded in paraffin. Using a rotary microtome, 4-μm coronal sections of the joints were cut and stained with Safranin O for histopathological analysis.

Histological evaluations were performed on PIP joint specimens to assess bone resorption and cartilage damage using Safranin O staining. PIP joints from the index, middle, ring, and little digits of both forelimbs and hind limbs were collected from each cynomolgus monkey, yielding a total of 16 PIP joints per animal. Each PIP joint was independently evaluated for inflammatory cell infiltration, pannus formation, cartilage lesion, bone resorption, and osteophyte formation using an ordinal semiquantitative grading system (0 to 4). To avoid pseudoreplication arising from multiple joints sampled from the same animal, joint-level scores were summarized at the animal level by calculating the mean score per animal before statistical analysis.

All experimental data are presented as mean ± SEM. For NHP studies, longitudinal clinical parameters were analyzed using the Kruskal-Wallis test with Dunn’s post hoc correction, as appropriate. For radiographic and histopathological analyses in the NHP model, statistical analyses were performed at the animal level. Comparisons between the EVT-401–treated and vehicle-treated groups were conducted using the Mann-Whitney *U* test. *P* < 0.05 was considered statistically significant.

### The humanized cartilage–RA SF coimplantation model in SCID mice

To investigate the capacity of primary RA SF to invade human cartilage in vivo, cultured human primary RA SF were harvested by trypsinization, suspended in sterile saline, and adjusted to a final volume of 100 μl per implant. Cartilage was obtained from nonarthritic donors undergoing knee surgery for traumatic injury, cut into 5- to 8-mm^3^ fragments, and preserved at −80°C until use. Six-week-old NOD-SCID mice (Cyagen Biosciences) served as recipients. On the day of transplantation, cartilage pieces were inserted into preincised sponge cubes (≈80 mm^3^), which were subsequently saturated with 4 × 10^5^ RA SF in saline. Each mouse received a subcutaneous implant containing both fibroblasts and cartilage and placed bilaterally under anesthesia and sterile conditions. Subcutaneous administration of 100 μM EVT-401 or vehicle control (0.1% DMSO) was performed every other day. After 4 weeks, implants were harvested, fixed in 4% paraformaldehyde, paraffin embedded, and sectioned for H&E staining. The extent of RA SF penetration into cartilage was graded using a semiquantitative scale: 0 = none or minimal; 1 = shallow (≤2 cell layers); 2 = moderate (≤5 cell layers); 3 = deep (>10 cell layers). Histological evaluation was carried out by two blinded independent observers.

### Statistical analysis

Statistical analyses were performed using the GraphPad Prism 8 software (GraphPad Software Inc., La Jolla, CA, USA). Data are presented as mean ± SEM unless otherwise specified. Correlations between two distinct parameters were analyzed using Spearman’s rank correlation test. The *r* and corresponding *P* values are shown in the plots. The Shapiro-Wilk test was used to assess data normality, and Levene’s test was used to assess homogeneity of variances. For normally distributed data with equal variances, parametric tests (Student’s *t* test for two groups and one-way or two-way ANOVA for multiple groups) were used. For data not meeting these assumptions, nonparametric tests (Mann-Whitney *U* test for two groups and Kruskal-Wallis test for multiple groups) were applied. When multiple comparisons were performed, *P* values were adjusted using the Benjamini-Hochberg FDR procedure. A two-sided *P* < 0.05 was considered statistically significant.

## Supplementary Materials

### The PDF file includes:

Supplementary data for characteristics of EVT-401 Figs. S1 to S8 Tables S1 to S7 Legends for data files S1 to S8
