# Supplementary material for: P2X7R of synovial fibroblasts is a potential therapeutic target associated with refractory rheumatoid arthritis
Source: Sci Adv. 2026 May 22;12(21):eadw9543. doi: 10.1126/sciadv.adw9543 (PMC13196751; doi:10.1126/sciadv.adw9543)
Supplement: Supplementary file 1 — Supplementary data for characteristics of EVT-401 Figs. S1 to S8 Tables S1 to S7 Legends for data files S1 to S8 [file sciadv.adw9543_sm.pdf]

Science Advances
AAAS

# Supplementary Materials for

## P2X7R of synovial fibroblasts is a potential therapeutic target associated with refractory rheumatoid arthritis

Peishi Rao *et al.*

Corresponding author: Qingping Jin, jinqp@conbapharm.com; Qingwen Wang, wangqingwen@pkuszh.com; Ming Chu, famous@bjmu.edu.cn; Xiaolin Sun, sunxiaolin_sxl@126.com; Zhanguo Li, li99@bjmu.edu.cn

*Sci. Adv.* **12**, eadw9543 (2026)
DOI: 10.1126/sciadv.adw9543

**The PDF file includes:**

Supplementary data for characteristics of EVT-401
Figs. S1 to S8
Tables S1 to S7
Legends for data files S1 to S8

**Other Supplementary Material for this manuscript includes the following:**

Data files S1 to S8

**Supplementary data for characteristics of EVT-401**

**The synthetic route and purification of EVT-401 (fig. S4)**

EVT-401 was a patented antagonist of human P2X7R licensed to Zhejiang Jinhua Conba Bio-Pharm. Co.,Ltd. by Renovis Inc. (ZL200780017026.5, PCT/US2007/006735 2007.03.16). Starting from a nitrobenzoate derivative, an enamine intermediate is first synthesized, which subsequently undergoes cyclization. The resulting compound then reacts with D-alaninol to afford a new intermediate, which is subjected to a Friedel–Crafts reaction. This is followed by successive decarboxylation steps using trifluoroacetic acid and potassium carbonate to yield a methylated intermediate. Acetyl protection is then introduced with acetic anhydride to obtain intermediate 5. Catalytic hydrogenation with palladium on carbon affords (R)-2-(6-methyl-5-amino-1-oxo-1,2-dihydroisoquinolin-2(1H)-yl)propanoate (intermediate 6), which is subsequently coupled with 3-fluoro-4-(trifluoromethyl)phenylacetic acid to produce 2-(3-fluoro-4-trifluoromethylphenyl)-N-[2-((R)-2-acetoxy-1-methyl-ethyl)-6-methyl-1-oxo-1,2-dihydroisoquinolin-5-yl]acetamide. Deacetylation under basic conditions yields EVT-401, which can be purified using ethyl acetate to afford pharmaceutical-grade EVT-401.

***in vitro*** **stability of EVT-401 (table S3)**

Supplementary Table 3 provides detailed *in vitro* stability data for EVT-401, demonstrating chemical (related substances, assay) and physical (appearance, drying loss) stability under accelerated/long-term conditions. The data supported EVT-401's formulation robustness, providing EVT-401 stability details.

**Pharmacokinetic parameters of EVT-401 (table S4 to S6)**

Following a single oral administration of EVT-401 at different doses (10 mg/kg, 30 mg/kg, and 100 mg/kg) in cynomolgus monkeys, the drug exhibited relatively slow absorption and elimination. Both Cmax and $AUC_{0\text{–}t}$ increased with rising doses. With dose ratios of 1:3:10, the corresponding Cmax ratios were 1:2.21:6.09, and $AUC_{0\text{–}t}$ ratios were 1:2.21:6.33. Linear regression analysis between the administered dose and both Cmax and $AUC_{0\text{–}t}$ yielded $R^2$ values of 1.00 and 0.99, respectively, indicating that EVT-401 exhibits linear pharmacokinetics in cynomolgus monkeys within the studied dose range (10–100 mg/kg).

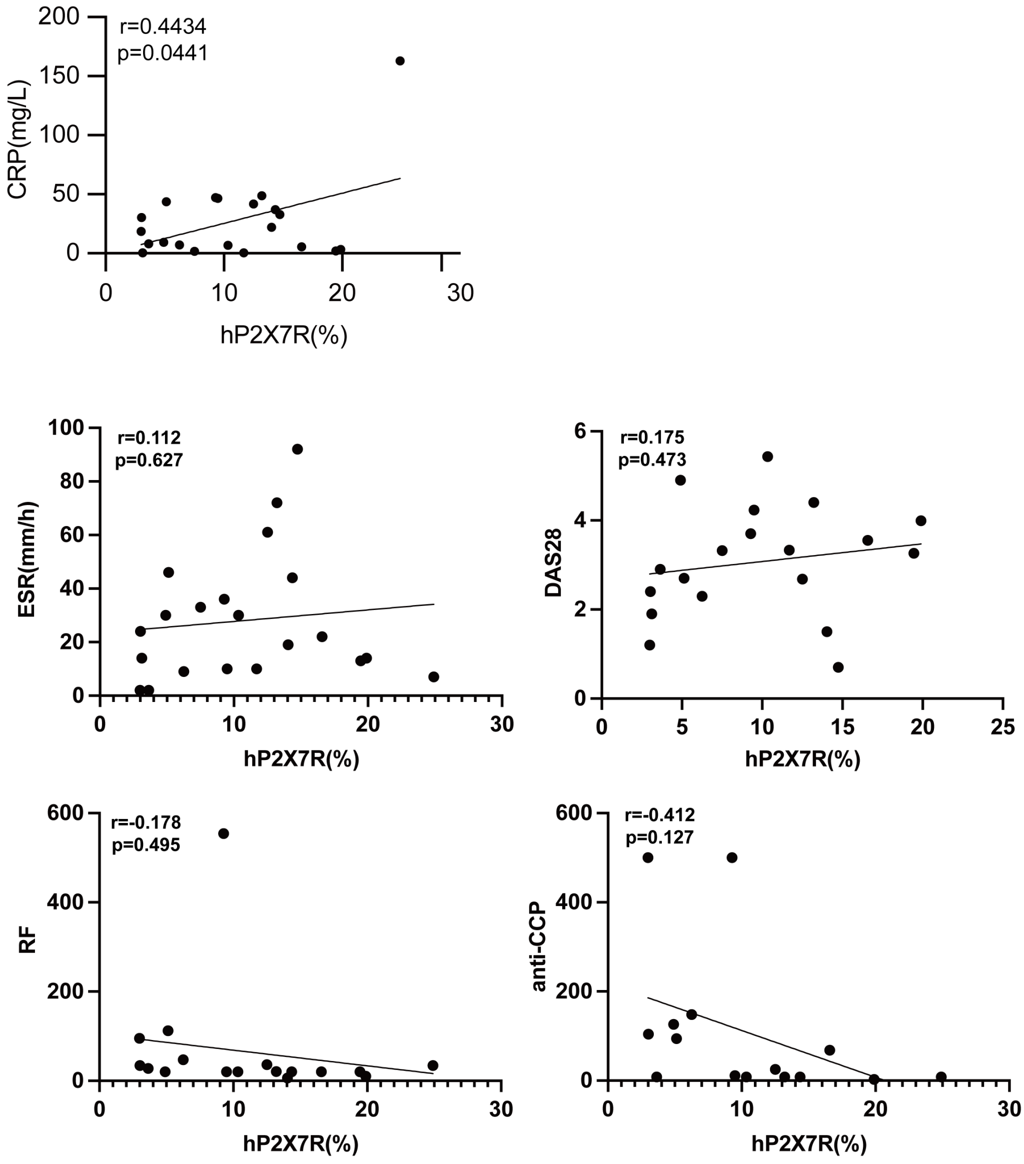


**Supplementary Fig. 1. The correlation between hP2X7R expression and clinical parameters.** The level of hP2X7R with different clicincal indicatiors, including C-reactive protein (CRP)， erythrocyte sedimentation rate (ESR), rheumatoid factor (RF), DAS28 score, and anti-cyclic citrullinated peptide antibody (anti-CCP), was analyzed using Spearman rank correlation.

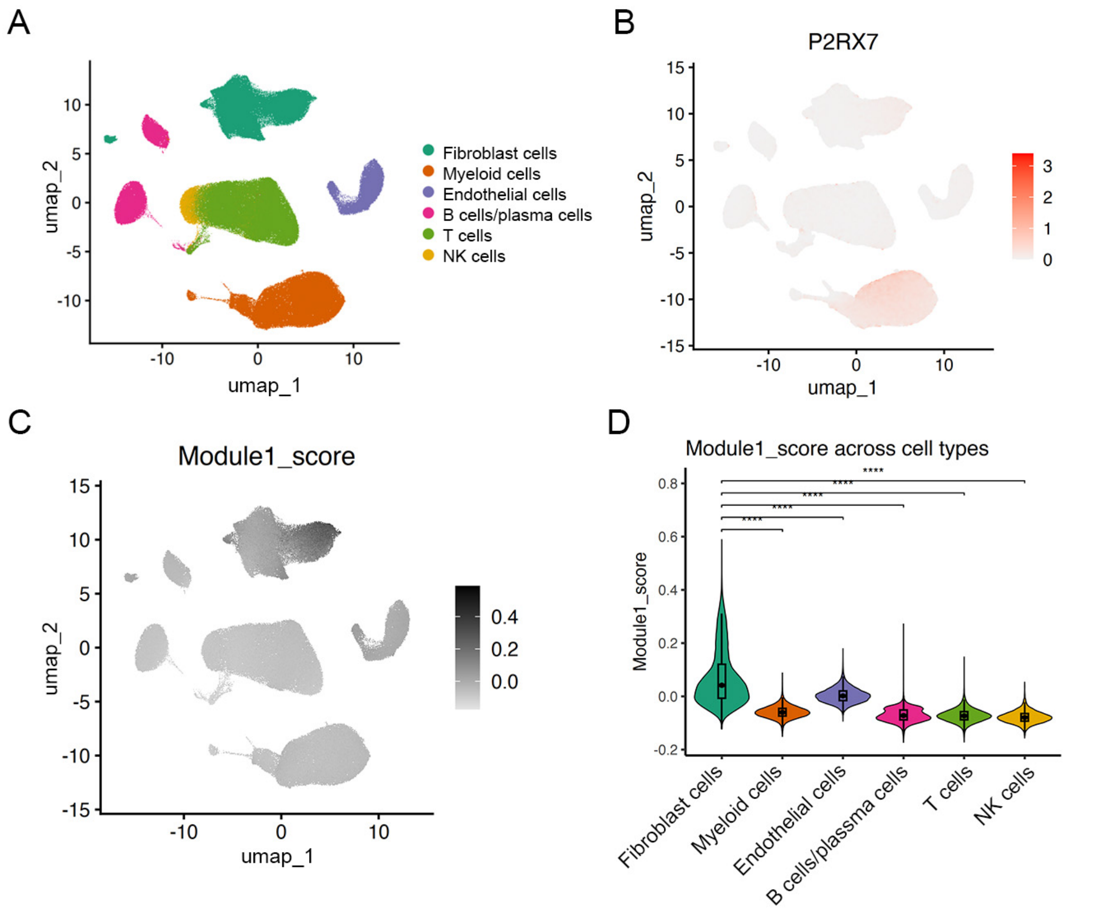


**Supplementary Fig. 2. Single-cell transcriptomic analysis of RA synovium highlightings *P2RX7* expression and Module1 activity.** (**A**) UMAP visualization of synovial single-cell transcriptomes from RA patients, identifying six major cell types: fibroblast cells, myeloid cells, endothelial cells, B/plasma cells, T cells, and NK cells. (**B**) UMAP feature plot illustrating *P2RX7* expression across cell types. (**C**)UMAP visualization of Module1 activity scores, showing preferential enrichment in fibroblasts. (**D**) Violin plot comparing Module1 activity scores across cell types, revealing significantly higher activity in fibroblasts compared with all other populations (Wilcoxon test, BH-adjusted, ****adjusted $p < 0.0001$).

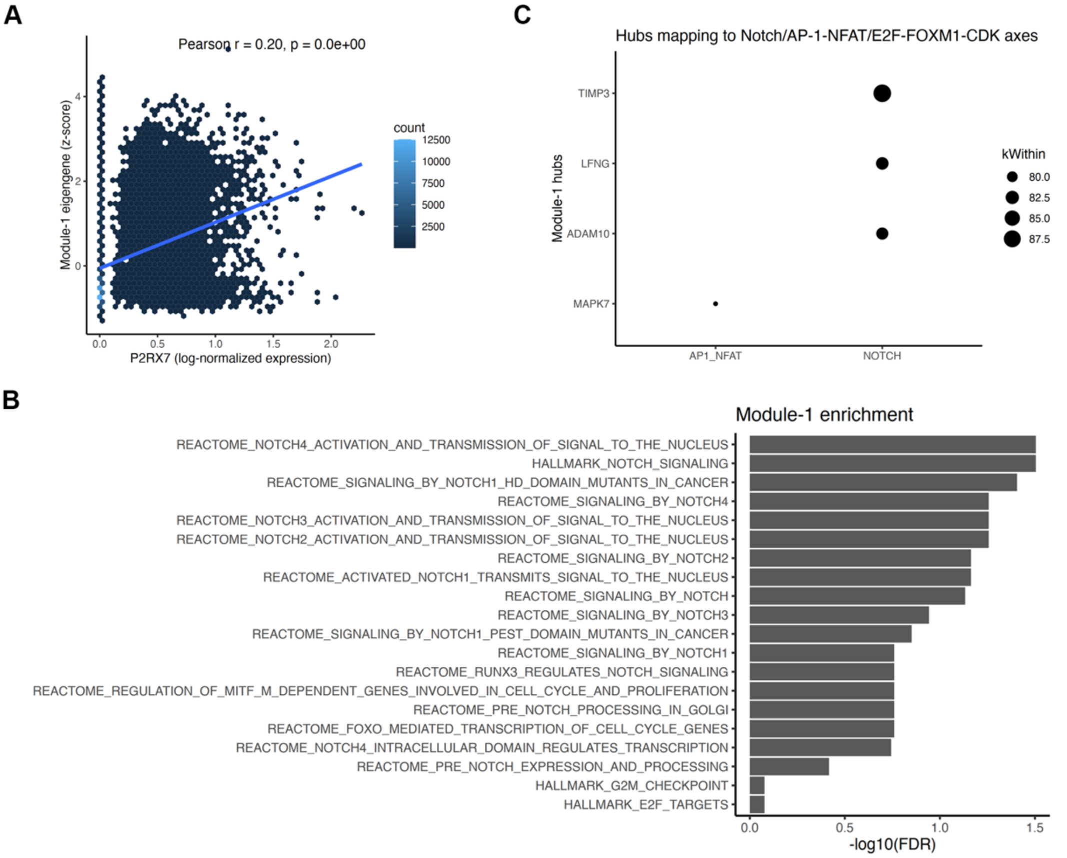


**Supplementary Fig. 3. *P2RX7*-linked Module-1 programs converge on Notch and cell-cycle signaling axes.** (**A**). Single-cell association between P2RX7 and Module-1 activity. The y-axis shows the Module-1 eigengene (PC1, z-score) computed from Module-1 genes; the x-axis shows log-normalized P2RX7 expression. Hexagons indicate cell density (color scale), and the blue line is the least-squares fit; the inset reports Pearson's r and two-sided p-value；(**B**). Functional enrichment of Module-1 genes. Bar plot shows significantly enriched pathways ranked by -log10(FDR). Notch-related pathways dominate, with additional enrichment for E2F, FOXO, and cell-cycle–associated signatures; (**C**) Module-1 hubs mapped to signaling axes. Bubbles represent Module-1 hub genes that overlap curated NOTCH (processing/tuning: ADAM10, TIMP3, LFNG) or AP-1/NFAT (e.g., MAPK7) gene sets. The x-axis indicates the axis; the y-axis lists hub genes; bubble size encodes within-module connectivity (kWithin). Only overlapping hubs are shown.

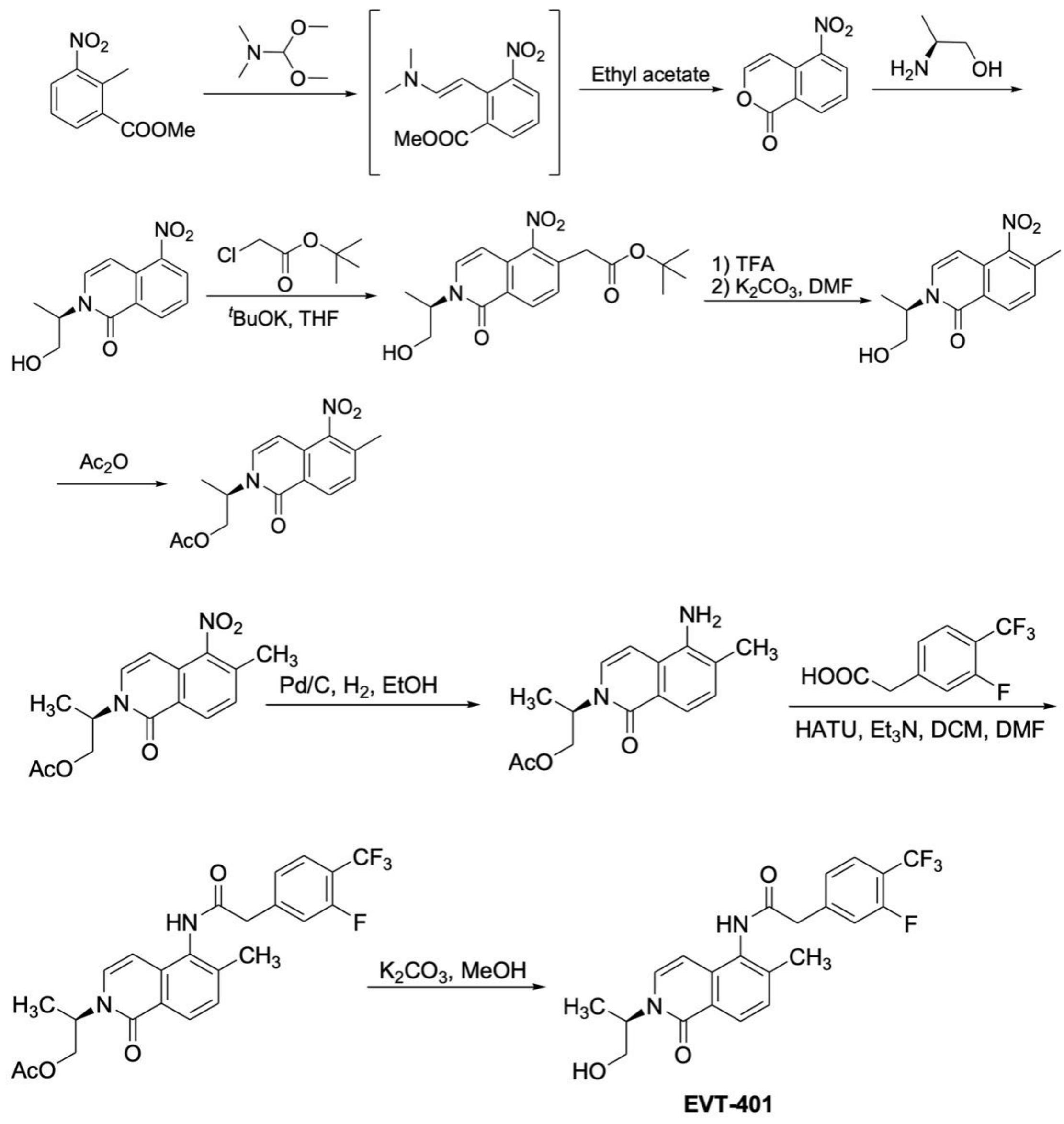


**Supplementary Fig. 4. The synthetic route of EVT-401.**

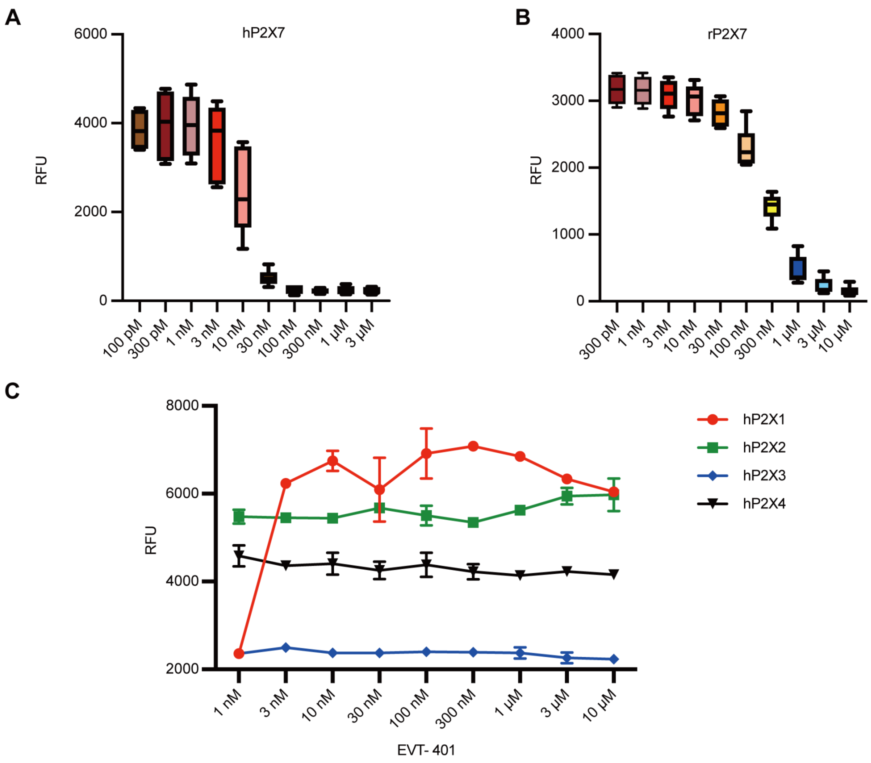


**Supplementary Fig. 5. Evaluation of EVT-401 in 1321N1 cells expressing human and rat P2X receptors through calcium influx measurement.** (**A,B**) EVT-401 effectively inhibited calcium influx in 1321N1 cells expressing human P2X7R (n=6) and rat P2X7R (n=8) , respectively. (**C**) EVT-401 showed no inhibition of ATP-induced calcium influx through P2X1R, P2X2R, P2X3R, or P2X4R (n=2).

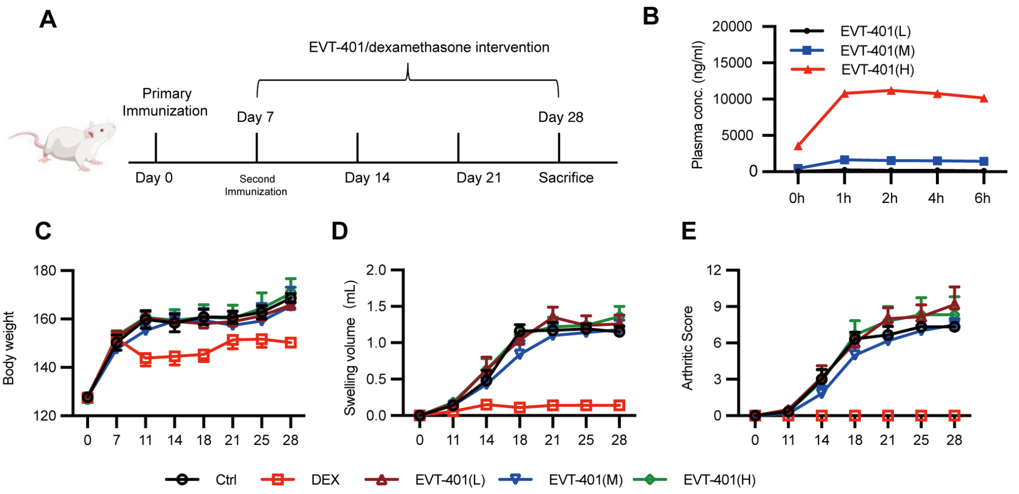


**Supplementary Fig. 6. Effect of EVT-401 on Lewis CIA rats.** (**A**)Experimental timeline of immunization and treatment. (B) Blood samples were collected to measure plasma concentrations. (**C-E**) Body weight, paw volume increase, and clinical arthritis scores were assessed according to the experimental protocol. n = 6 in each group.EVT-401(L):0.5mg/kg, EVT-401(M):5mg/kg, EVT-401(H):50mg/kg

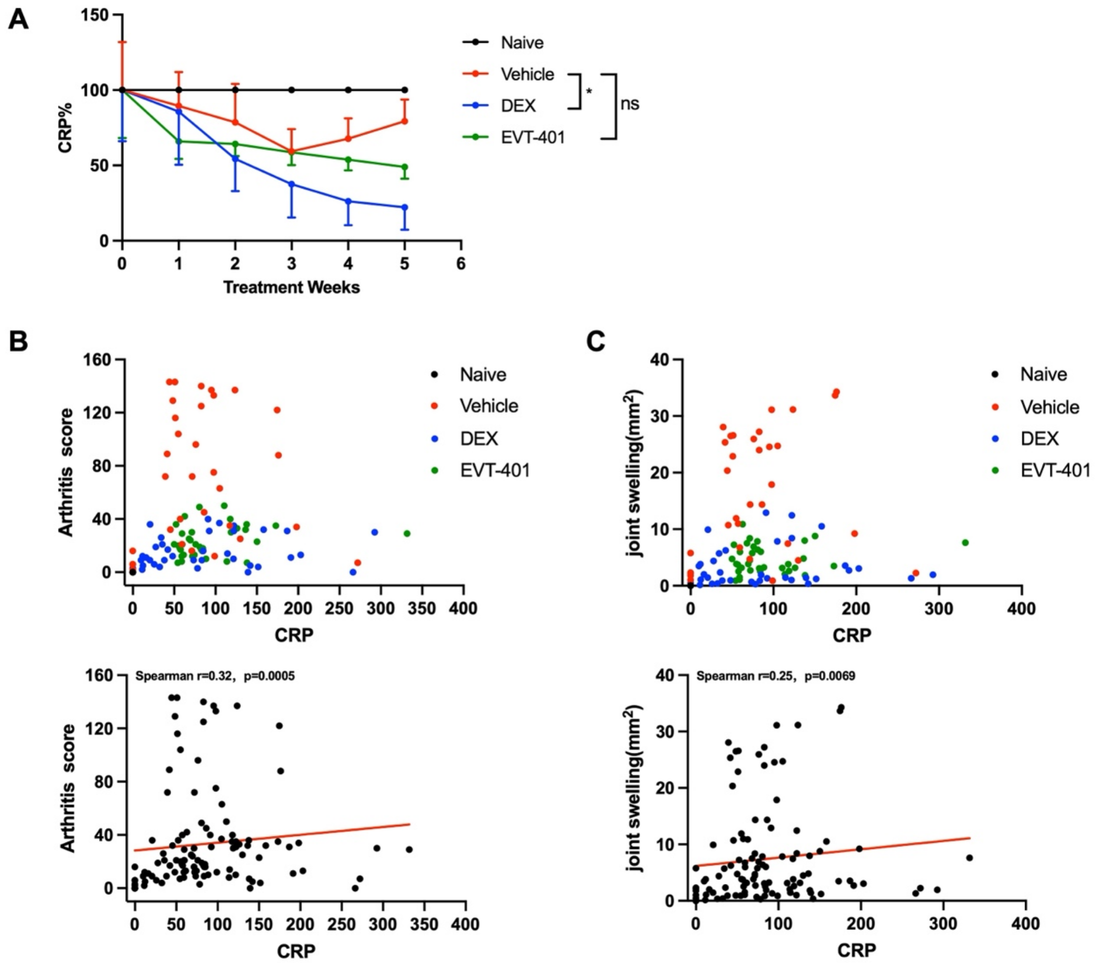


**Supplementary Fig. 7.Serum CRP levels reflect treatment response and correlate with joint inflammation in NHPs.** (**A**) Longitudinal measurement of serum CRP concentrations (mean ± SEM) in different treatment groups over 6 weeks. (**B**) Correlation between serum CRP and arthritis score across all animals and timepoints. Each dot represents one animal at a specific timepoint; colors denote treatment groups. (**C**) Correlation between serum CRP and joint swelling area. CRP levels positively correlated with tissue swelling severity (Spearman r = 0.25, p = 0.008). Correlations between two parameters were analyzed using Spearman's rank correlation test. The correlation coefficient (r) and corresponding p values are shown in the plots.

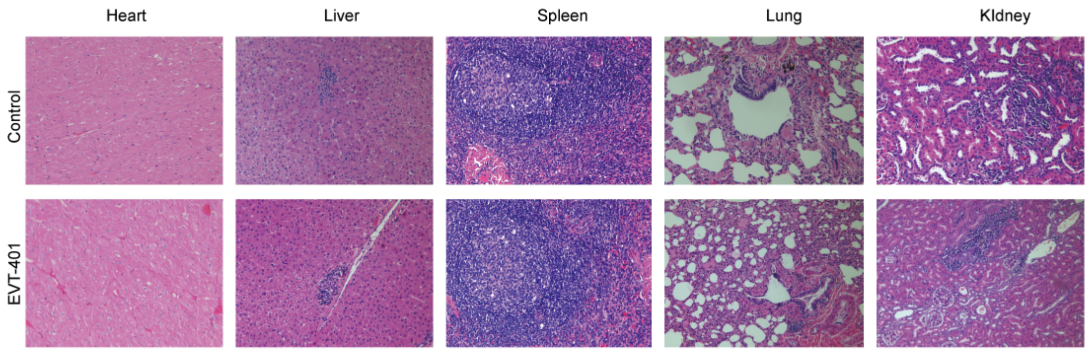


**Supplementary Fig. 8. Histological analysis of organ damage in different treatment groups.** H&E staining of the heart, liver, spleen, lungs, and kidneys was performed to assess organ damage across the treatment groups. (Scale bars, 100 µm)

**Supplementary Table 1. Baseline Clinical Characteristics of Patients Included in the Cohort for IHC and H&E Analysis (n=22).**

| | |
|---|---|
| **Demographics** | |
| Age, mean ± SD, years | 55.4±7.3 |
| Female Gender, n (%) | 17(77.2%) |
| Disease duration, mean ± SD, years | 6.6±7.2 |
| **Biochemical and clinical parameters** | |
| RF+, n (%) | 13（63.6%） |
| Anti-CCP+, n (%) | 11（50.0%） |
| ESR, mm/h, mean ± SD | 27.6±23.7 |
| CRP, mg/L, mean ± SD | 26.3±35.2 |
| DAS28, mean ± SD | 3.2±1.3 |
| **Biopsy Location** | |
| Knee, n (%) | 20(90.9%) |
| Hip, n (%) | 2(9.1%) |

Abbreviations: n, number; SD, standard deviation; RF, rheumatoid factor; CCP, cyclic citrullinated peptide; ESR, erythrocyte sedimentation rate; CRP, C-reactive protein; DAS28, Disease Activity Score 28.

**Supplementary Table 2. The specific Krenn scores of synovial samples from different RA patients in our cohort.**

| RA patient (n=22) | synovial linging cell layer | density of the resident cells | inflammatory infiltrate | Krenn score | Group |
|---|---|---|---|---|---|
| RA patient 1 | 0 | 0 | 0 | 0 | No synovitis |
| RA patient 2 | 0 | 1 | 0 | 1 | No synovitis |
| RA patient 3 | 0 | 0 | 0 | 0 | No synovitis |
| RA patient 4 | 0 | 0 | 1 | 1 | No synovitis |
| RA patient 5 | 0 | 0 | 1 | 1 | No synovitis |
| RA patient 6 | 0 | 0 | 0 | 0 | No synovitis |
| RA patient 7 | 0 | 0 | 0 | 0 | No synovitis |
| RA patient 8 | 0 | 0 | 0 | 0 | No synovitis |
| RA patient 9 | 0 | 1 | 0 | 1 | No synovitis |
| RA patient 10 | 1 | 1 | 1 | 3 | Low-grade synovits |
| RA patient 11 | 1 | 1 | 2 | 4 | Low-grade synovits |
| RA patient 12 | 1 | 1 | 1 | 3 | Low-grade synovits |
| RA patient 13 | 1 | 1 | 0 | 2 | Low-grade synovits |
| RA patient 14 | 2 | 1 | 0 | 3 | Low-grade synovits |
| RA patient 15 | 0 | 2 | 2 | 4 | Low-grade synovits |
| RA patient 16 | 3 | 0 | 0 | 3 | Low-grade synovits |
| RA patient 17 | 1 | 1 | 1 | 3 | Low-grade synovits |
| RA patient 18 | 3 | 1 | 2 | 6 | High-grade synovits |
| RA patient 19 | 1 | 2 | 3 | 6 | High-grade synovits |
| RA patient 20 | 1 | 2 | 3 | 6 | High-grade synovits |
| RA patient 21 | 1 | 2 | 2 | 5 | High-grade synovits |
| RA patient 22 | 1 | 2 | 2 | 5 | High-grade synovits |

**Supplementary Table 3. In vitro stability data of EVT-401 under accelerated and long-term conditions.**

| Item | Storage conditions | Testing Time Points | Testing items | Analytical Methods and Their Validation |
|---|---|---|---|---|
| Accelerated Testing | Temperature 40°C±2°C, Relative Humidity 75%±5% | 0, 1, 2, 3, 6 months | Appearance, related substances, isomers, loss on drying, assay, microbial limit* | Validated and included in the quality standard |
| Long-term Testing | Temperature 25°C±2°C, Relative Humidity 60%±5% | 0,3,6,9,12,18,24,36 months | Appearance, related substances, isomers, loss on drying, assay, microbial limit* | Validated and included in the quality standard |
| Conclusion | EVT-401 remains stable under accelerated and long-term conditions for 24 months, with all parameters meeting specifications. | | | |

**Supplementary Table 4. Mean plasma concentration–time profile of EVT-401 following oral administration in cynomolgus monkeys**

| Time(h) | plasma drug concentration (ng/mL, Mean±SD)) | | |
|---|---|---|---|
| | 10mg/kg (n=6) | 30mg/kg (n=6) | 100mg/kg (n=6) |
| 0 | 0 | 0 | 0 |
| 0.25 | 103.58 ± 66.10 | 397.16 ± 367.19 | 570.60 ± 772.56 |
| 0.5 | 200.50 ± 84.57 | 565.79 ± 340.50 | 697.93 ± 722.87 |
| 1 | 250.98 ± 72.01 | 688.44 ± 274.03 | 1021.25 ± 1021.12 |
| 2 | 321.59 ± 104.22 | 693.89 ± 194.77 | 1345.74 ± 1140.71 |
| 4 | 329.86 ± 155.79 | 477.06 ± 192.45 | 1859.35 ± 800.54 |
| 6 | 208.67 ± 70.49 | 521.07 ± 288.15 | 2080.45 ± 782.96 |
| 8 | 256.23 ± 79.88 | 449.65 ± 261.88 | 1637.98 ± 982.72 |
| 10 | 220.76 ± 63.18 | 429.35 ± 220.00 | 1401.49 ± 783.41 |
| 24 | 76.95 ± 39.39 | 198.21 ± 92.41 | 491.59 ± 141.93 |
| 30 | 44.97 ± 23.55 | 112.31 ± 39.27 | 235.71 ± 90.65 |
| 48 | 20.04 ± 3.18 | 26.65 ± 10.82 | 85.95 ± 13.18 |

**Supplementary Table 5. Pharmacokinetic parameters of EVT-401 following oral administration in cynomolgus monkeys**

| Parameters | Unit | PO-10mg/kg | PO-30mg/kg | PO-100mg/kg |
|---|---|---|---|---|
| $AUC_{(0-t)}$ | μg/L*h | 5407.69 ± 1206.41 | 11940.18 ± 3381.07 | 34246.4±12110.99 |
| $MRT_{(0-t)}$ | h | 11.65 ± 2.97 | 13.49 ± 2.26 | 12.61±1.52 |
| $T_{1/2}$ | h | 10.56 ± 6.36 | 12.86 ± 10.28 | 9.87±3.31 |
| Tmax | h | 3.00 ± 1.10 | 2.29 ± 2.27 | 5.67±2.34 |
| CLz/F | L/h/kg | 1.76 ± 0.39 | 2.46 ± 0.73 | 3.15±1.31 |
| Vz/F | L/kg | 26.39 ± 16.38 | 42.66 ± 28.49 | 45.94±25.46 |
| Cmax | μg /L | 403.51 ± 108.62 | 891.24 ± 177.46 | 2457.04±951.50 |

Abbreviation: $AUC_{0}$–t: Area Under the Plasma Concentration–Time Curve from time zero to last measurable concentration; MRT: Mean Residence Time; $T_{1/2}$: Elimination Half-life; Tmax: Time to Maximum Plasma Concentration; CLz/F: Apparent Clearance after Oral Administration; Vzz/F: Apparent Volume of Distribution after Oral Administration; Cmax: Maximum Plasma Concentration

**Supplementary Table 6. Bioavailability of EVT-401 in cynomolgus monkeys**

| Dosage | PO-10mg/kg | PO-30mg/kg | PO-100mg/kg |
|---|---|---|---|
| $AUC_{0\text{-}t}$(μg/L*h) | 5407.7±1206.4 | 11940.2±3381.07 | 34246.4±12110.99 |
| %, bioavailability | (42.2±9.4) % | (30.9±8.8) % | (26.6±9.4) % |

**Supplementary Table 7. EVT-401 inhibited calcium influx in 1321N1 cells**

| Rat P2X7 IC50(nM) (n=4) | Human P2X7 IC50(nM) (n=3) | Human P2X1 IC50(nM) (n=1) | Human P2X2 IC50(nM) (n=1) | Human P2X3 IC50(nM) (n=1) | Human P2X4 IC50(nM) (n=1) |
|---|---|---|---|---|---|
| 248 | 9.6 | >10000 | >10000 | >10000 | >10000 |
| 257 | 6.7 | | | | |
| 175 | 13.6 | | | | |
| 200 | | | | | |

**Supplemental Data**

**Supplementary data.1. All genes of the regulatory network Module 1** (excel file)

**Supplementary data.2. Comparative analysis of sequence homology between rat and human P2X7 receptors (P2X7R)**(excel file)

**Supplementary data.3.Upregulated genes in EVT-401 group compared to TNF-α group** (excel file)

**Supplementary data.4. Downregulated genes in EVT-401 group compared to TNF-α group** (excel file)

**Supplementary data.5. Upregulated genes in TNF-α group compared to RA SF group**(excel file)

**Supplementary data.6. Downregulated genes in TNF-α group compared to RA SF group** (excel file)

**Supplementary data.7. Raw radiographic scores for each individual DIP, PIP and MCP joint from each Cynomolgus monkey**(excel file)

**Supplementary data.8. Raw histopathological scores for each individual PIP joint from each Cynomolgus monkey**(excel file)
